# Decoupling speciation and extinction reveals both abiotic and biotic drivers shaped 250 million years of diversity in crocodile-line archosaurs

**DOI:** 10.1038/s41559-023-02244-0

**Published:** 2023-12-04

**Authors:** Alexander R. D. Payne, Philip D. Mannion, Graeme T. Lloyd, Katie E. Davis

**Affiliations:** 1 Department of Biology, University of York, York, UK; 2 Leverhulme Centre for Anthropocene Biodiversity, University of York, York, UK; 3 Department of Earth Sciences, University College London, London, UK; 4 Independent Researcher, Amble, UK

**Keywords:** Phylogenetics, Biodiversity, Speciation, Palaeontology

## Abstract

Whereas living representatives of Pseudosuchia, crocodylians, number fewer than 30 species, more than 700 pseudosuchian species are known from their 250-million-year fossil record, displaying far greater ecomorphological diversity than their extant counterparts. With a new time-calibrated tree of >500 species, we use a phylogenetic framework to reveal that pseudosuchian evolutionary history and diversification dynamics were directly shaped by the interplay of abiotic and biotic processes over hundreds of millions of years, supported by information theory analyses. Speciation, but not extinction, is correlated with higher temperatures in terrestrial and marine lineages, with high sea level associated with heightened extinction in non-marine taxa. Low lineage diversity and increased speciation in non-marine species is consistent with opportunities for niche-filling, whereas increased competition may have led to elevated extinction rates. In marine lineages, competition via increased lineage diversity appears to have driven both speciation and extinction. Decoupling speciation and extinction, in combination with ecological partitioning, reveals a more complex picture of pseudosuchian evolution than previously understood. As the number of species threatened with extinction by anthropogenic climate change continues to rise, the fossil record provides a unique window into the drivers that led to clade success and those that may ultimately lead to extinction.

## Abstract (web summary)

Using a new phylogeny of Pseudosuchia (crocodile-line archosaurs), the authors use diversification analyses and information theory to show that the interplay of abiotic and biotic processes over hundreds of millions of years shaped evolutionary history and diversification dynamics in this clade.

## Main

Global temperature, atmospheric CO_2_, ocean acidification and sea level are all predicted to continue to rise^1^, and projected climate scenarios could effectively reverse as much as 50 million years of long-term cooling^2^. These anthropogenically driven environmental changes are already exerting a profound effect on extant biodiversity, with rates of extinction approaching those of the ‘big five’ mass extinctions of the geological past^3^, and new biotic interactions resulting from climatically driven^4^ and human-mediated^5^ geographic shifts in species ranges. Yet, through geological time, the diversity of life on Earth has always been shaped by changes in the physical environment^6^ and/or by fluctuations in biotic interactions^7^. In reality, it is likely that some combination of these abiotic and biotic factors is responsible for the diversification of many clades^8,9^. The evolutionary history of clade diversification can therefore provide crucial insights into the long-term impact of anthropogenically driven changes to the environment and biosphere on extant biodiversity.

Pseudosuchia is a clade of archosaurian reptiles, defined as all species more closely related to crocodylians than to birds^10^. Extant pseudosuchians are all members of Crocodylia, a group of semi-aquatic ambush predators found predominantly in freshwater habitats of the tropics^11^. Of the 25–27 extant species of Crocodylia currently recognized, seven are categorized as Critically Endangered, with a further four species identified as vulnerable, with declining populations^12^. Many species reside in low-lying areas, meaning that rising sea levels associated with global warming may irreversibly alter the habitats on which they depend^13^. Although extant pseudosuchians have low species richness, more than 700 extinct species are currently recognized in the fossil record^14–19^. They first appear in the fossil record shortly after the Permian/Triassic mass extinction, 252 million years ago (Ma), and evolved to occupy a variety of habitats and niches, including large terrestrial carnivores, heavily armoured herbivores and fully marine forms^10,20–24^.

Extant Pseudosuchia is represented by very limited ecomorphological diversity compared to their extinct representatives, yet their closest living relatives, birds (Aves), have diversified to approximately 11,000 extant species^25^, showing a vast array of ecomorphological diversity^26^. This asymmetry in the fate of sister clades is a well-documented macroevolutionary phenomenon^27–30^, but how it arises is not well understood. In both Pseudosuchia and Aves, climate has been proposed as a major driver of diversity^15,31–40^. Modern birds radiated rapidly in the wake of the Cretaceous/Paleogene mass extinction, 66 Ma^41,42^, and continued to diversify throughout the Cenozoic^43^—an era characterized by a general long-term cooling trend^44^. However, pseudosuchians were at their most diverse during periods of global warming^15,35^, with evidence for declining diversification correlated with the Cenozoic long-term global cooling trend^15,34^. The relative contribution of biotic factors is less evident^22,39^. A recent study provided an attempt to tease apart the relative roles of biotic and abiotic drivers of diversification dynamics of Crocodylia over the past 100 million years^45^. These authors found evidence that net diversification of crocodylians over macroevolutionary timescales has likely been shaped by both biotic and abiotic factors.

In this Article, we go further by evaluating the relative roles of abiotic and biotic factors on the entire evolutionary history of pseudosuchian diversification dynamics. We test the effects of environmental change and clade competition, via proxies, throughout the group’s 250 million year evolutionary history with a time-calibrated phylogeny comprising more than 500 species. We demonstrate that pseudosuchian evolutionary history was shaped by the interplay of ecological niche with both biotic and abiotic processes over hundreds of millions of years, supported by a direct transfer of information from our abiotic and biotic time series to speciation and extinction rates.

## Results

### Pseudosuchian phylogeny

**Fig. 1:**
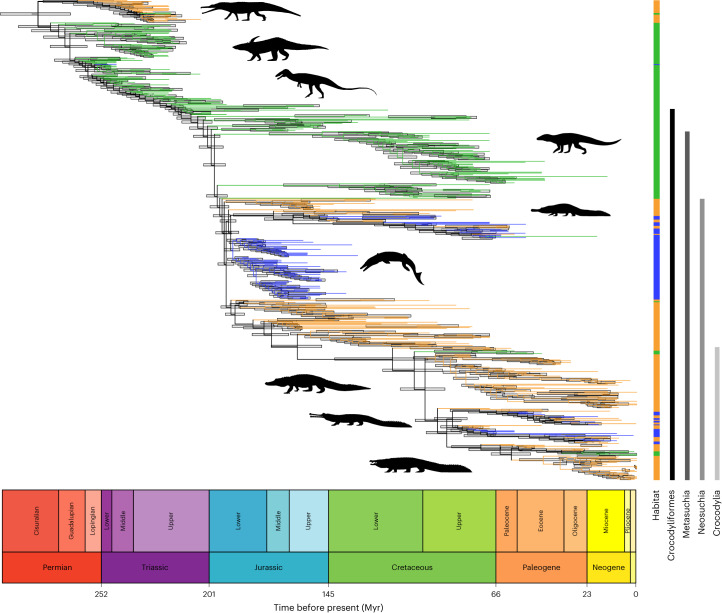
Time-calibrated supertree of Pseudosuchia. Maximum agreement subtree of 534 taxa, scaled to geological time. Terminal branches are colour coded according to ecology (blue, marine; green, terrestrial; orange, freshwater), and light grey node bars represent node age ranges (95% highest posterior density). Ecologies were mapped to branches using IToL v.6.8.1^126^. Taxa highlighted by silhouettes from PhyloPic (phylopic.org) to showcase pseudosuchian morphological disparity are (from top to bottom): Phytosauria, Aetosauria, Poposauroidea, Notosuchia, Tethysuchia, Thalattosuchia, Alligatoroidea, Gavialoidea and Crocodyloidea. Silhouettes from S. Hartman, D. Bogdanov, N. Tamura and M. Keesey are licensed under CC BY 3.0; A. Reindl licensed under CC BY 4.0; and F. Sayol, S. Traver and Jagged Fang Designs under CC0 1.0 Universal. The geological timescale was added using the R package ‘strap’ v.1.6-0^127^.

The resultant metatree (Fig. 1) contains 534 taxa and, to our knowledge, is the most inclusive pseudosuchian phylogeny published. The overall topology is broadly consistent with recent Crocodylomorpha supertrees^22^. The root of the tree is placed in the Permian, 282 Ma (95% interval, 266–299 Ma), with Crocodylomorpha and Crocodyliformes estimated to have originated in the Middle Triassic (240 Ma; 95% interval, 235–247 Ma) and Late Triassic (214 Ma; 95% interval, 210–219 Ma), respectively. Neosuchia is estimated to have originated in the Early Jurassic (195 Ma; 95% interval, 191–199 Ma), which is broadly consistent with previous estimates^22,46^. Crocodylia diverged in the mid-Cretaceous, 100 Ma (95% interval, 90–111 Ma), which is also consistent with recent studies^47,48^. Within Crocodylia, Alligatoroidea is recovered outside of the Crocodyloidea + Gavialoidea clade, reflecting the relationships of molecular, but not most morphological, analyses^48,49^.

### Diversification dynamics through time

**Fig. 2:**
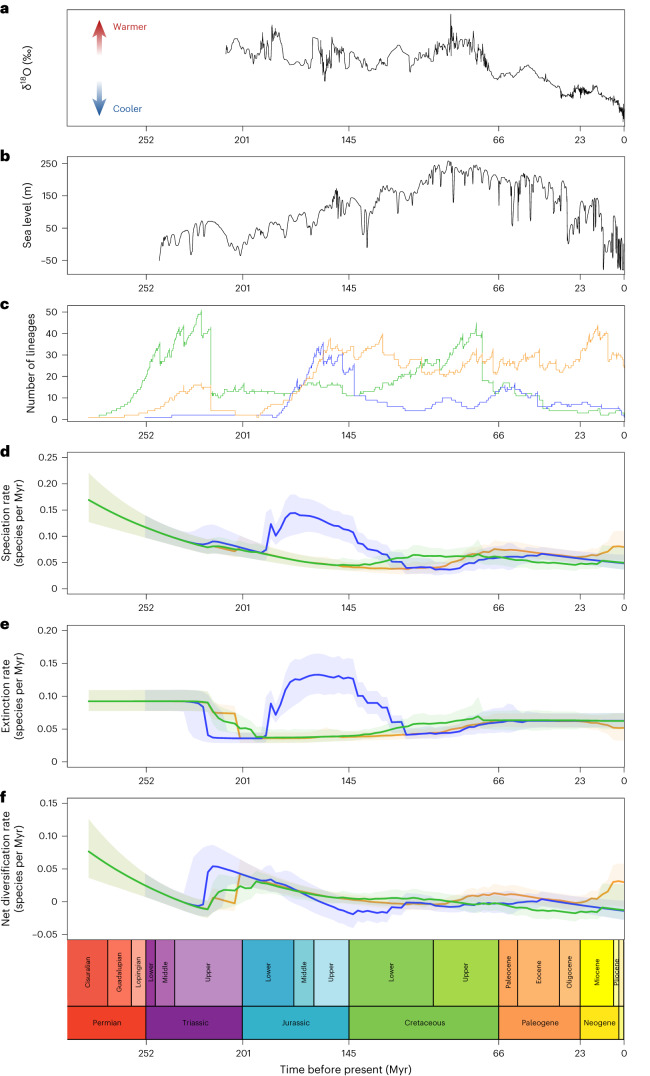
Abiotic and biotic time series and pseudosuchian diversification dynamics. **a**, Global temperature; **b**, eustatic sea level; **c**, lineage diversity; **d**, speciation rates; **e**, extinction rates; **f**, net diversification rates. All are scaled to geological time along the *x* axis. Panels **c**–**f** are colour coded according to ecology (blue, marine; green, terrestrial; orange, freshwater). In **d**–**f**, the solid line represents the mean of 9,001 realizations of the diversification rate through time, while lighter shading represents the 95% CI. Diversification dynamics were plotted with the R package ‘BAMMtools’ v.2.1.10^106^, LTT were plotted with the R package ‘ape’ v.5.7-1^113^, and the geological timescale was added using the R package ‘strap’ v.1.6-0^127^.

Exclusively terrestrial pseudosuchians experienced their highest levels of diversity during the Triassic (Fig. 2), followed by a sharp decline over the Triassic/Jurassic boundary, 201 Ma, with only the crocodylomorph lineage surviving^50^. This is followed by low levels of diversity, speciation and extinction throughout the Jurassic and Early Cretaceous. Pseudosuchians regained high levels of diversity in the Late Cretaceous, following a period of heightened speciation rates that marked the notosuchian radiation^51^. Towards the end of the Cretaceous, terrestrial pseudosuchians experienced a sharp diversity decline, which continued across the Cretaceous/–Paleogene boundary, with only sebecosuchian notosuchians surviving the mass extinction^52^. Diversity generally declined throughout the Cenozoic, with sebecosuchians disappearing in the Middle Miocene^53^. The last exclusively terrestrial group, the mekosuchine crocodyloids that were endemic to Oceania and first appeared in the fossil record in the early Eocene^54^, survived until the Holocene, only going extinct sometime in the past 4,000 years^55^.

Freshwater pseudosuchians reached an initial diversity peak during the Triassic, followed by a sharp decline at the Triassic/Jurassic boundary (Fig. 2). They subsequently radiated, reaching a peak in the Late Jurassic and Early Cretaceous. Although remaining generally high, diversity of freshwater species was volatile throughout the Cretaceous with generally high speciation, extinction and net diversification across the Cretaceous/Paleogene mass extinction event. During the Cenozoic, the highest diversity of freshwater species is observed during the Miocene, comprising members of Crocodylia^15,40,56,57^. Subsequently, numbers declined to that of present-day diversity.

The first major marine invasion followed the Triassic/Jurassic mass extinction (Fig. 2), with the rapid radiation of thalattosuchian crocodyliforms^58,59^, following which speciation rate, extinction rate and diversity all reached their highest levels by the Middle Jurassic. Thalattosuchians then experienced a sharp decrease in diversity and speciation and extinction rate at the end of the Jurassic. Although they do not return to Middle Jurassic levels, both speciation/extinction rates and lineage diversity in the marine realm increased during the Cretaceous (Fig. 2), mainly driven by the independent radiation of tethysuchians^16^, with an additional diversification of gavialoids in the latest Cretaceous^60,61^. Speciation rates, extinction rates and overall diversity were unaffected by the Cretaceous/Paleogene mass extinction, with both tethysuchians (primarily dyrosaurids) and gavialoids surviving^16,61^. Marine diversity reached another peak early in the Paleogene, driven primarily by gavialoids, including taxa traditionally regarded as early diverging tomistomines^48^. Subsequently, diversity and diversification rates were low, but relatively stable, in the marine realm (Fig. 2), before the extinction of all remaining fully marine lineages during the Plio-Pleistocene interval.

### Abiotic and biotic correlations

**Fig. 3:**
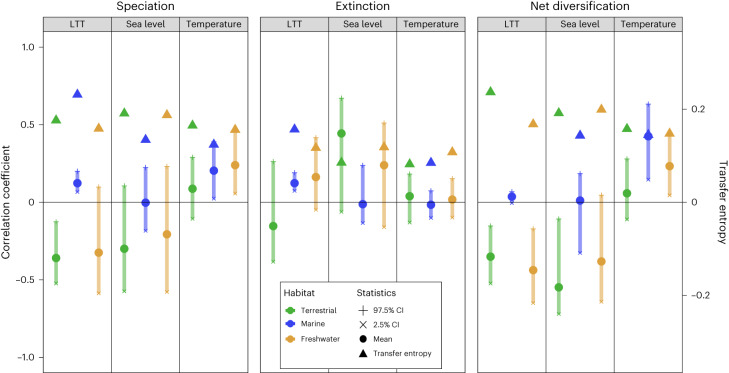
Time series correlations and transfer entropy results. Results for all correlations showing the mean, 2.5% and 97.5% CIs, and transfer entropy results for each habitat partition for each time series (blue, marine; green, terrestrial; orange, freshwater). *N* = 9,001 independent samples as derived from the diversification rate analyses. All correlations are significant at *P* < 2.2 × 10^−16^ as assessed with a Wilcoxon signed-rank test, while all transfer entropy values are significant at *P* < 0.001 as assessed by a Markov block boot strap^121^.

Overall, our results show that all three variables tested, global temperature, global sea level and lineages through time (LTT), influenced pseudosuchian diversification; however, the effects are not homogenous across ecologies (see Fig. 3 and Table 1 for full results). Here we only consider results that recovered mean correlation coefficient values of greater than ±0.1 as all were statistically significant (*P* < 2.2 × 10^−16^).

**Table 1:** Time series correlations and transfer entropy results

		Speciation				Extinction				Net diversification			
		Mean	2.5% CI	97.5% CI	Transfer entropy	Mean	2.5% CI	97.5% CI	Transfer entropy	Mean	2.5% CI	97.5% CI	Transfer entropy
**Marine**	**Sea level**	−0.003	−0.184	0.224	0.134	−0.012	−0.134	0.237	NA	0.011	−0.327	0.184	0.143
	**Temperature**	*0.204*	*0.024*	*0.390*	*0.124*	−0.016	−0.101	0.074	0.085	*0.425*	*0.146*	*0.633*	*0.145*
	**LTT**	*0.123*	*0.067*	*0.200*	*0.231*	*0.123*	*0.074*	*0.190*	*0.157*	0.036	−0.005	0.068	NA
**Terrestrial**	**Sea level**	−*0.207*	−*0.579*	*0.231*	*0.187*	*0.239*	−*0.160*	*0.511*	*0.118*	*−0.382*	−*0.642*	*0.046*	*0.199*
	**Temperature**	*0.239*	*0.056*	*0.463*	*0.156*	0.017	−0.099	0.152	0.108	*0.233*	*0.045*	*0.438*	*0.148*
	**LTT**	−*0.325*	−*0.587*	*0.098*	*0.158*	*0.162*	−*0.048*	*0.416*	*0.117*	−*0.438*	−*0.650*	−*0.171*	*0.168*
**Freshwater**	**Sea level**	−*0.300*	−*0.574*	*0.105*	*0.191*	*0.444*	−*0.062*	*0.671*	*0.085*	*−0.549*	−*0.720*	−*0.108*	*0.192*
	**Temperature**	0.087	−0.107	0.290	0.165	0.039	−0.131	0.182	0.082	0.058	−0.111	0.279	0.158
	**LTT**	−*0.360*	−*0.524*	−*0.126*	*0.176*	−*0.154*	−*0.383*	*0.262*	*NA*	−*0.351*	−*0.524*	−*0.153*	*0.237*
**Non-marine**	**Sea level**	−*0.317*	−*0.604*	*0.121*	*0.182*	*0.329*	−*0.073*	*0.584*	*0.134*	−*0.517*	−*0.698*	−*0.114*	*0.184*
	**Temperature**	0.090	−0.106	0.306	0.160	0.055	−0.104	0.209	0.113	0.043	−0.131	0.268	0.152
	**LTT**	−*0.525*	−*0.696*	−*0.224*	*0.185*	−*0.253*	−*0.512*	*0.125*	*0.131*	−*0.470*	−*0.662*	−*0.215*	*0.208*

Mean, 2.5% and 97.5% CIs and transfer entropy results are given for each habitat partition for each time series. All correlations are significant at *P* < 2.2 × 10^−16^, while all reported transfer entropy values are significant at *P* < 0.001. NA means that no significant transfer entropy values were found at that CI. Correlations with a correlation coefficient of greater than ±0.1 are italicized.

We find that warmer temperatures are associated with increased speciation in both marine and terrestrial lineages, with a strong positive correlation for both partitions, but do not recover any relationship with temperature in freshwater lineages. There is no evidence for a relationship between temperature and extinction rates in any of the ecological partitions. The interaction of speciation and extinction rates results in increased net diversification in both marine and terrestrial lineages with increasing global temperatures.

Lower sea levels are associated with increased speciation in terrestrial and freshwater lineages, whereas higher sea levels are associated with extinction in both these ecologies. There is no relationship recovered between sea level and either speciation or extinction for marine lineages. This results in a decrease in net diversification with higher sea levels for terrestrial and freshwater lineages.

For our LTT analyses, fewer numbers of lineages are strongly associated with increased speciation in terrestrial and freshwater ecologies, whereas increased numbers of lineages have a weak positive effect on marine lineages. Extinction shows a different pattern, with increased numbers of lineages associated with increased extinction in terrestrial and marine lineages, with the reverse observed in freshwater lineages; that is, fewer numbers of lineages is correlated with higher extinction rates in these ecologies. Net diversification decreases with increased numbers of lineages for terrestrial and freshwater lineages, whereas lineage diversity does not show any correlation with net diversification for marine lineages.

Due to the difficulties in confidently assigning some non-marine taxa to either terrestrial or freshwater habitats, we also analysed these categories together in a ‘non-marine’ partition. For this combined partition, we find no evidence for a relationship between temperature and speciation or extinction rates; however, we recover a negative correlation between sea level and speciation rates and a positive correlation between sea level and extinction rates. Net diversification is strongly negatively correlated with sea level. This partition is also strongly and negatively correlated with speciation, extinction and net diversification rates for LTT.

### Information transfer

For all but two of our analyses that indicate that one of our three variables is a driver of diversification, our transfer entropy results reveal the presence of information transfer (we considered our results to show some evidence of information transfer if they returned values >0.1; see Table 1). These exceptions are both found in freshwater lineage extinction rates—the first is sea level, which shows a positive correlation, but a low value (0.09) for information transfer. The second is LTT, which shows a negative correlation, but no significant results are returned for information transfer. Otherwise, our results are strongly congruent with the strength of the correlation (as measured by mean rate). All reported information transfer results are significant at *P* < 0.001, and only statistically significant results were retained (see Table 1 for full results).

### Accounting for phylogenetic and temporal uncertainty

**Extended Data Fig. 1:**
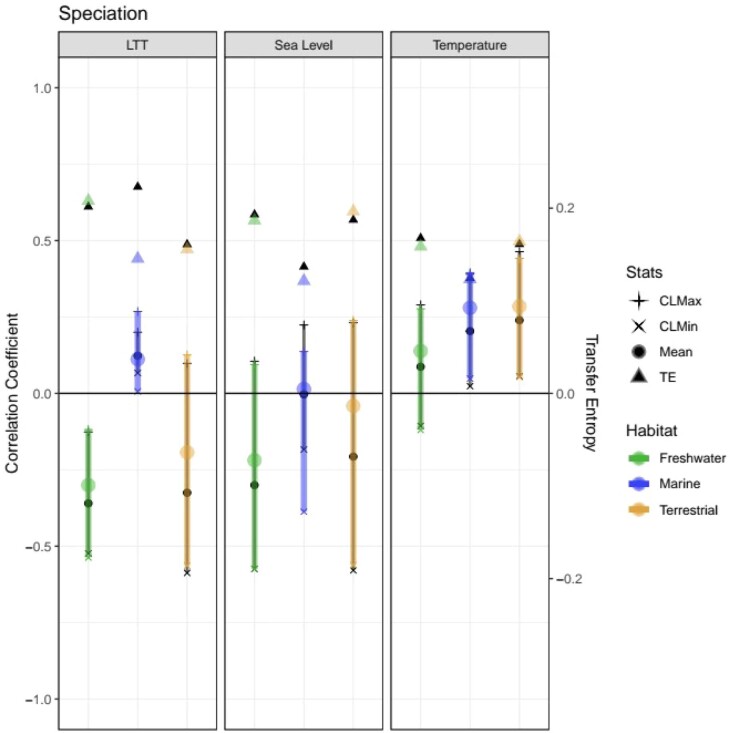
Time series correlations and transfer entropy results for speciation analyses with Thalattosuchia as sister to Neosuchia. Results for all correlations showing the mean, 2.5% and 97.5% confidence intervals, and Transfer Entropy (TE) results for each habitat partition for each time series (marine = blue, terrestrial = green, freshwater = orange). N = 9,001 independent samples as derived from the diversification rate analyses. All correlations are significant at p < 2.2e-16 as assessed with a Wilcoxon signed-rank test, while all transfer entropy values are significant at p < 0.001 as assessed by a Markov block boot-strap^121^.

**Extended Data Fig. 2:**
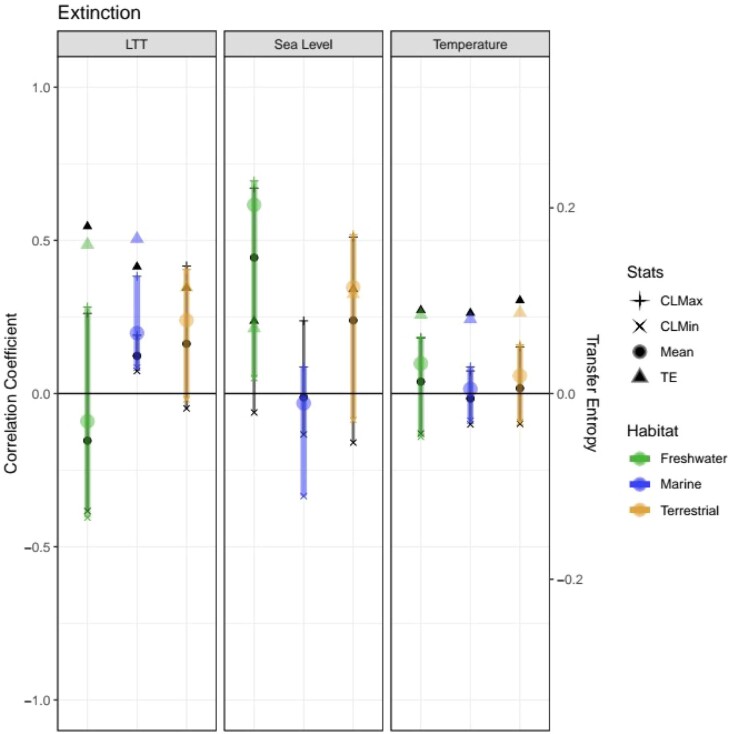
Time series correlations and transfer entropy results for extinction analyses with Thalattosuchia as sister to Neosuchia. Results for all correlations showing the mean, 2.5% and 97.5% confidence intervals, and Transfer Entropy (TE) results for each habitat partition for each time series (marine = blue, terrestrial = green, freshwater = orange). N = 9,001 independent samples as derived from the diversification rate analyses. Coloured symbols represent results from sensitivity analyses, black symbols represent results from the Maximum Clade Credibility tree. All correlations are significant at p < 2.2e-16 as assessed with a Wilcoxon signed-rank test, while all transfer entropy values are significant at p < 0.001 as assessed by a Markov block boot-strap^121^.

**Extended Data Fig. 3:**
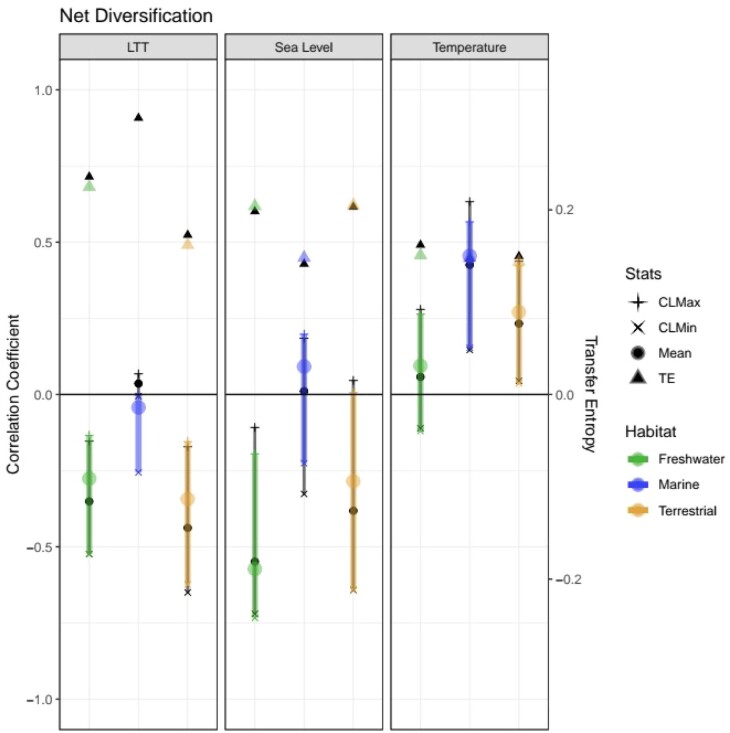
Time series correlations and transfer entropy results for net diversification analyses with Thalattosuchia as sister to Neosuchia. Results for all correlations showing the mean, 2.5% and 97.5% confidence intervals, and Transfer Entropy (TE) results for each habitat partition for each time series (marine = blue, terrestrial = green, freshwater = orange). N = 9,001 independent samples as derived from the diversification rate analyses. Coloured symbols represent results from sensitivity analyses, black symbols represent results from the Maximum Clade Credibility tree. All correlations are significant at p < 2.2e-16 as assessed with a Wilcoxon signed-rank test, while all transfer entropy values are significant at p < 0.001 as assessed by a Markov block boot-strap^121^.

**Extended Data Fig. 4:**
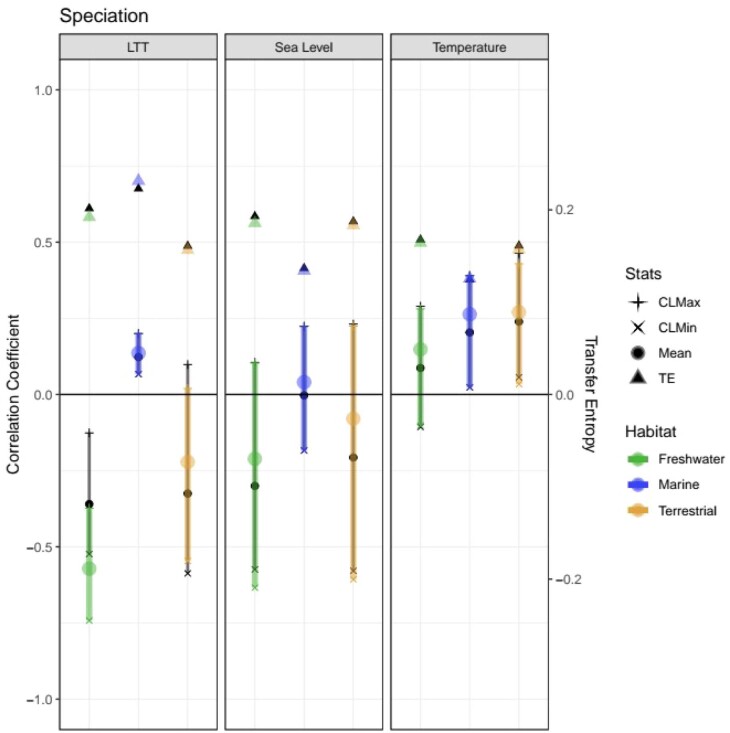
Time series correlations and transfer entropy results for speciation analyses with Phytosauria excluded. Results for all correlations showing the mean, 2.5% and 97.5% confidence intervals, and Transfer Entropy (TE) results for each habitat partition for each time series (marine = blue, terrestrial = green, freshwater = orange). N = 9,001 independent samples as derived from the diversification rate analyses. Coloured symbols represent results from sensitivity analyses, black symbols represent results from the Maximum Clade Credibility tree. All correlations are significant at p < 2.2e-16 as assessed with a Wilcoxon signed-rank test, while all transfer entropy values are significant at p < 0.001 as assessed by a Markov block boot-strap^121^.

**Extended Data Fig. 5:**
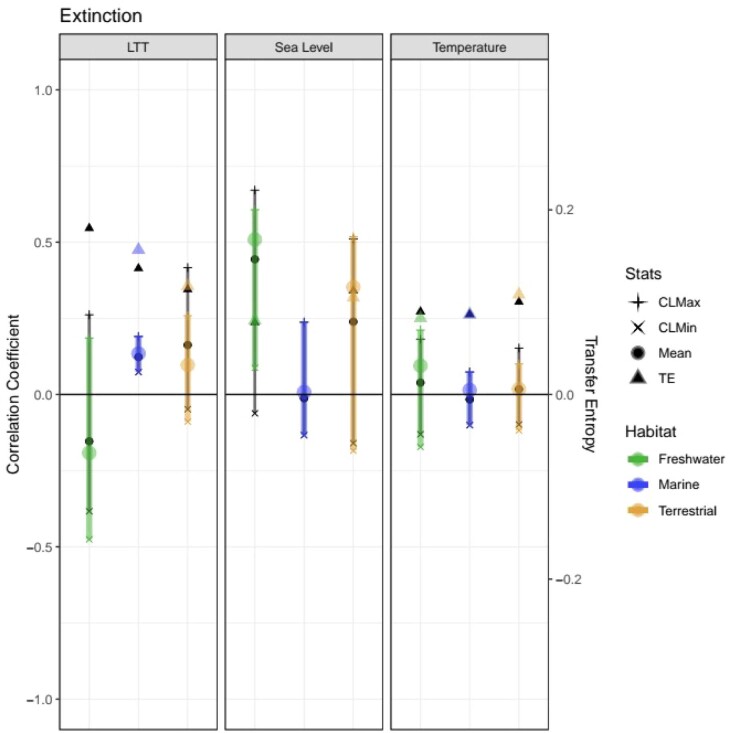
Time series correlations and transfer entropy results for extinction analyses with Phytosauria excluded. Results for all correlations showing the mean, 2.5% and 97.5% confidence intervals, and Transfer Entropy (TE) results for each habitat partition for each time series (marine = blue, terrestrial = green, freshwater = orange). N = 9,001 independent samples as derived from the diversification rate analyses. Coloured symbols represent results from sensitivity analyses, black symbols represent results from the Maximum Clade Credibility tree. All correlations are significant at p < 2.2e-16 as assessed with a Wilcoxon signed-rank test, while all transfer entropy values are significant at p < 0.001 as assessed by a Markov block boot-strap^121^.

**Extended Data Fig. 6:**
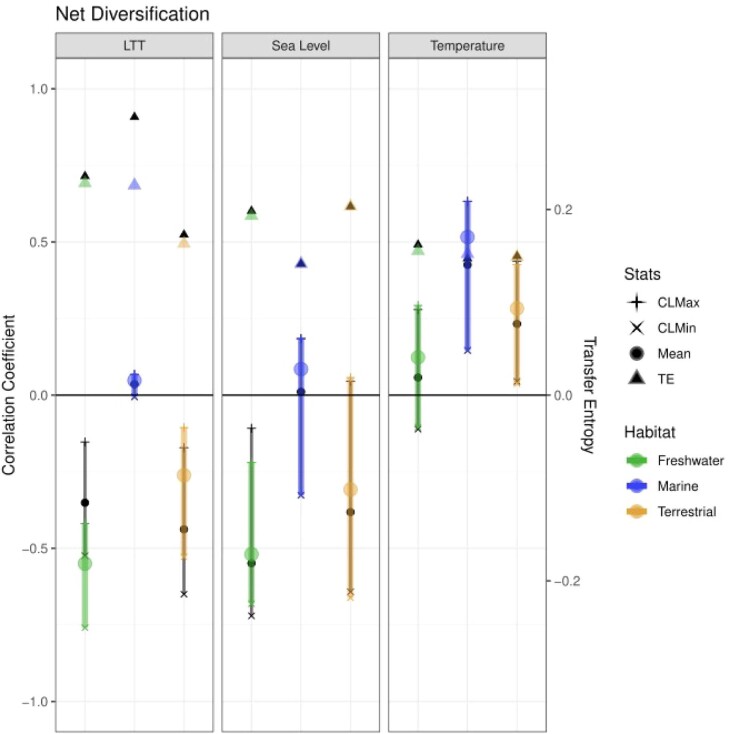
Time series correlations and transfer entropy results for net diversification analyses with Phytosauria excluded. Results for all correlations showing the mean, 2.5% and 97.5% confidence intervals, and Transfer Entropy (TE) results for each habitat partition for each time series (marine = blue, terrestrial = green, freshwater = orange). N = 9,001 independent samples as derived from the diversification rate analyses. Coloured symbols represent results from sensitivity analyses, black symbols represent results from the Maximum Clade Credibility tree. All correlations are significant at p < 2.2e-16 as assessed with a Wilcoxon signed-rank test, while all transfer entropy values are significant at p < 0.001 as assessed by a Markov block boot-strap^121^.

**Extended Data Fig. 7:**
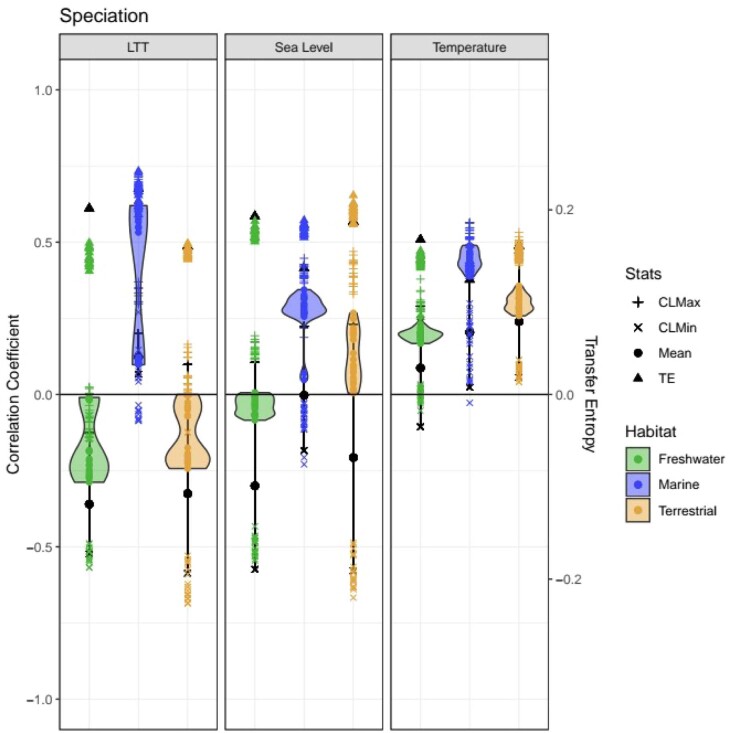
Time series correlations and transfer entropy results for speciation analyses assessing the impact of temporal uncertainty. Results for all correlations showing the mean, 2.5% and 97.5% confidence intervals, and Transfer Entropy (TE) results for each habitat partition for each time series (marine = blue, terrestrial = green, freshwater = orange). N = 9,001 independent samples as derived from the diversification rate analyses. Coloured symbols represent results from sensitivity analyses, black symbols represent results from the Maximum Clade Credibility tree. Violin plots represent the distribution of results from 20 trees from the posterior distribution. All correlations are significant at p < 2.2e-16 as assessed with a Wilcoxon signed-rank test, while all transfer entropy values are significant at p < 0.001 as assessed by a Markov block boot-strap^121^.

**Extended Data Fig. 8:**
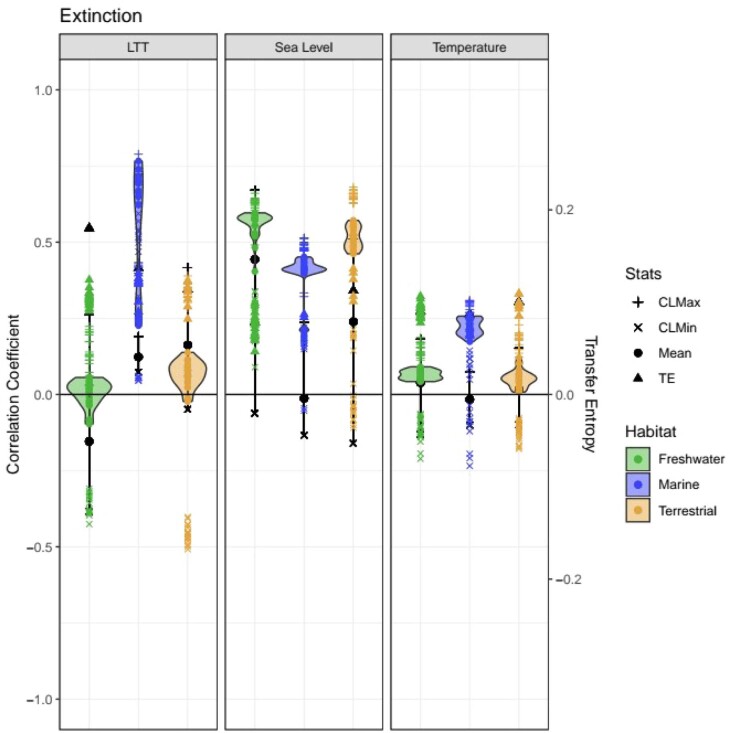
Time series correlations and transfer entropy results for extinction analyses assessing the impact of temporal uncertainty. Results for all correlations showing the mean, 2.5% and 97.5% confidence intervals, and Transfer Entropy (TE) results for each habitat partition for each time series (marine = blue, terrestrial = green, freshwater = orange). N = 9,001 independent samples as derived from the diversification rate analyses. Coloured symbols represent results from sensitivity analyses, black symbols represent results from the Maximum Clade Credibility tree. Violin plots represent the distribution of results from 20 trees from the posterior distribution. All correlations are significant at p < 2.2e-16 as assessed with a Wilcoxon signed-rank test, while all transfer entropy values are significant at p < 0.001 as assessed by a Markov block boot-strap^121^.

**Extended Data Fig. 9:**
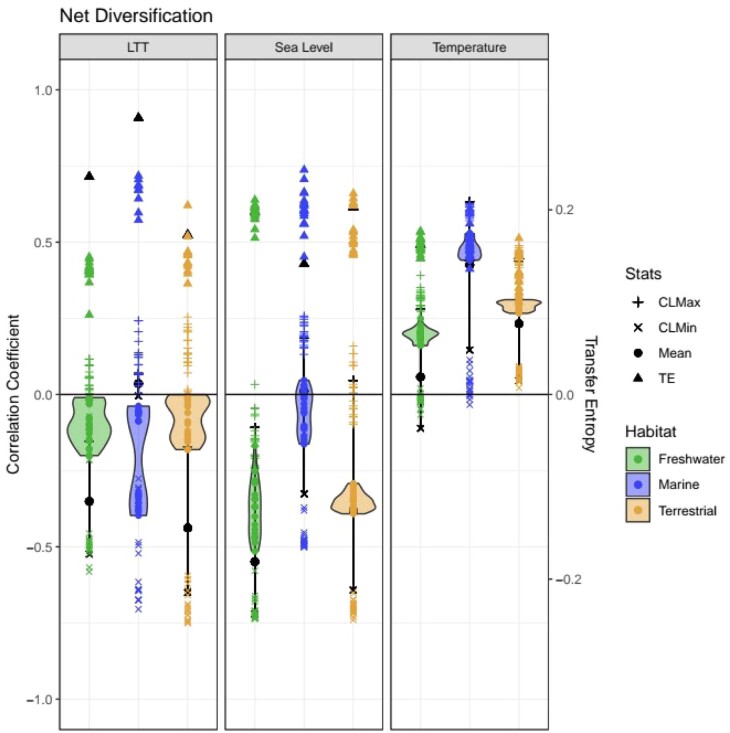
Time series correlations and transfer entropy results for net diversification analyses assessing the impact of temporal uncertainty. Results for all correlations showing the mean, 2.5% and 97.5% confidence intervals, and Transfer Entropy (TE) results for each habitat partition for each time series (marine = blue, terrestrial = green, freshwater = orange). N = 9,001 independent samples as derived from the diversification rate analyses. Coloured symbols represent results from sensitivity analyses, black symbols represent results from the Maximum Clade Credibility tree. Violin plots represent the distribution of results from 20 trees from the posterior distribution. All correlations are significant at p < 2.2e-16 as assessed with a Wilcoxon signed-rank test, while all transfer entropy values are significant at p < 0.001 as assessed by a Markov block boot-strap^121^.

We also tested two alternative phylogenetic hypotheses—Thalattosuchia as the sister clade to Crocodyliformes, rather than Neosuchia, and excluding Phytosauria from Pseudosuchia. Furthermore, we explored the effect of temporal uncertainty on our results. Phylogenetic uncertainty has no notable impact on our results, which are remarkably consistent regardless of the input phylogeny and in some instances yielded even stronger signals than our main results presented above. Temporal uncertainty has a more varied effect on the results, and the key differences are as follows: speciation in marine and terrestrial lineages is now positively correlated with sea level, whereas extinction in marine lineages is now positively correlated with sea level. Net diversification for these pairs of variables remains unchanged. Overall, our results remain unchanged by these sensitivity analyses. See Extended Data Figs. 1–9 for the full results of these analyses.

## Discussion

Temperature has long been recognized as a driver of biological turnover^6,62–64^. Here we show that pseudosuchian speciation rates are positively correlated with fluctuations in temperature for both marine and terrestrial ecologies, but not in freshwater taxa, whereas there is no clear relationship between temperature and extinction rate for any of our ecological partitions. The one exception to this might be marine lineages, which show a low positive correlation with extinction in our analyses that assess the impact of temporal variation. Previous work has found broadly congruent results, including a positive correlation between warmer temperatures and higher pseudosuchian diversity in general. However, all previous studies either have only evaluated taxonomic diversity or have not separated pseudosuchian taxa into these separate ecological categories^15,38,40,45,65^, whereas our study quantitatively shows the long-term effect of global warming on speciation rates in fully terrestrial pseudosuchians. Thermophysiology might explain why we see a positive correlation between temperature and speciation, with extant species ectothermic and characterized by a subtropical distribution, with heightened ecological sensitivity to ambient temperature^11,66,67^. Palaeohistological analyses indicate that although the thermophysiology of the earliest pseudosuchians was likely closer to that of endotherms^68^, the transition to ectothermy had occurred in the group at least by the time of the divergence of Metasuchia (the group comprising Neosuchia + Notosuchia)^69^, in the Early Jurassic. Although higher temperatures might have directly led to increased speciation rate, this also meant that more of the Earth was habitable for pseudosuchians^15,34^, with their fossils known from subpolar latitudes in the Eocene^70^. As such, the positive correlation between temperature and speciation rate might partly or primarily reflect a species-area effect, with higher speciation coincident with the latitudinal extension of the warm temperate climatic belt^45^. Speciation rate in freshwater pseudosuchians, however, does not appear to be related to fluctuations in global temperature. This is an unexpected finding, given that extant species are primarily freshwater^66,67^. One potentially confounding factor is the difficulty associated with assigning non-marine fossil taxa to either freshwater or terrestrial habitats. To account for this, we ran analyses with terrestrial and freshwater species combined into a non-marine category, but this also did not show any relationship between speciation rate and temperature. We therefore suggest that this absence of a correlation is a genuine result and not simply the result of difficulty assigning habitat states. One explanation could be that speciation in freshwater taxa is more closely tied to other environmental factors that we did not test, such as aridity^15,71,72^. A positive correlation between temperature and speciation rate and either no correlation, or a relatively smaller correlation, with extinction rate in marine pseudosuchians potentially reconciles conflicting results from previous studies that evaluated taxonomic diversity^15,38,73^, which is essentially a product of both rates.

We find that pseudosuchian speciation rates increase with lower sea levels in both terrestrial and freshwater lineages. Conversely, extinction rates for these lineages increase with higher sea levels, suggesting that sea level regressions led to increased speciation, whereas transgressions drove extinction. The net result of this is a negative correlation between net diversification and sea level for terrestrial pseudosuchians, which is in line with previous work^15^, in which either no relationship or a negative correlation was recovered between sea level and taxonomic diversity of non-marine pseudosuchians^39^. It is plausible that both terrestrial and freshwater taxa benefited from sea level regressions via the species area effect, whereby the creation of increased habitat availability allowed for increased diversification^73–75^. Conversely, marine transgressions might have led to higher rates of extinction in both terrestrial and freshwater lineages as a result of suitable habitat being lost during periods of continental flooding^73,76^. It is important to note that the results for speciation in terrestrial lineages were the most variable of our results when subjected to sensitivity analysis. Therefore, while our results are in line with previous work and our conclusions remain unchanged by our sensitivity analyses, we cannot state with confidence that the impact of sea level on decoupled speciation and extinction rates in terrestrial pseudosuchians can be clearly delineated.

A lack of correlation between diversification rates of marine pseudosuchians and sea level is surprising and also contrasts with previous studies that have tended to recover a positive correlation^38,39^, at least with taxonomic diversity. A positive correlation between sea level and speciation for marine lineages is recovered when temporal variation is taken into account; therefore, our results might still be congruent with previous work. However, these results are from a random sample of just 20 trees, and therefore the maximum clade credibility results may still be representative of the results as a whole. We are therefore cautious in drawing strong conclusions at this stage.

One limitation of our abiotic correlation tests is the use of global proxies. This approach assumes that there is no spatial variation in environmental parameters, but this is patently not the case, especially for palaeotemperature^77^. If spatially explicit palaeoenvironmental data were available, a better solution would be to partition both the biotic and abiotic data geographically to obtain a clearer picture of the effect that temperature had on diversification at regional scales. Nevertheless, we believe that our global-scale analyses are still useful in identifying the relative role played by environmental parameters in shaping pseudosuchian diversity over macroevolutionary timescales, even if more precise conclusions cannot currently be drawn.

Diversity dependence, as a proxy for intra-clade competition, drives both speciation and extinction across all three of our ecological partitions. Both terrestrial and freshwater lineages have higher speciation rates when lineage diversity is low. This suggests that low lineage diversity results in opportunities for niche filling, accomplished through increased speciation rates, similar to what we might see in an adaptive radiation^78–80^. The radiation of Neosuchia, following the end-Triassic mass extinction, might be an example of such niche filling^50^. For terrestrial taxa, extinction rates are positively correlated with lineage diversity, which suggests that increased competition with more lineages leads to elevated rates of extinction^8,80^. This might potentially characterize the rise of pseudosuchians during the Triassic^50^, as well as patterns of turnover in notosuchian faunas in the Cretaceous^51^. Freshwater lineages show the opposite pattern, with higher extinction rates corresponding to low lineage diversity. However, standard deviation is high and confidence intervals (CIs) broad for freshwater taxa, and we therefore cannot be confident that this result is biologically meaningful. In marine lineages, both speciation and extinction rates are higher with increased lineage diversity. High speciation rates and similarly high extinction rates can result from rapid turnover^81^, as the traits that lead to elevated speciation rates are often the same ones that lead to higher rates of extinction^82^. It is therefore plausible that competition, as a result of higher lineage diversity in marine taxa, stimulated speciation while also driving extinction, as lineages were out-competed. Our analyses do not differentiate between intra-clade and inter-clade competition; therefore, the signal recovered might result from competition between marine clades or between lineages within clades. Qualitatively, such a scenario potentially corresponds to the following macroevolutionary trajectory: thalattosuchians declined as tethysuchians first appeared, with dyrosaurid tethysuchians and gavialoids only appearing towards the end of the Cretaceous, when most non-dyrosaurid tethysuchians disappeared^16,60^. The surviving marine lineages appear to have thrived after the Cretaceous/Paleogene mass extinction, which has been generally attributed to the vacancy of ecospace^15,83^.

It is also possible that competition between pseudosuchians and other clades played a role in shaping their diversity. For example, terrestrial and freshwater pseudosuchians might have been in competition with some dinosaurs; similarly, marine pseudosuchians might have competed with plesiosaurs, ichthyosaurs and mosasaurs in the Mesozoic, cetaceans from the Eocene onwards, and with sharks since pseudosuchians first entered the marine realm. There are also other biotic factors at play that we have not considered here. One such factor is body size, with previous research showing that low body size disparity in crocodylians is associated with increased extinction risk^45^ and that body size evolution might also be linked to environmental change^22,65^. The full picture of the role played by biotic variables in pseudosuchian macroevolution is therefore undoubtedly far more complex than so far revealed.

Our transfer entropy analyses are strongly supportive of our correlation results, with a high level of congruence indicating the presence of information transfer from the abiotic and biotic variables to diversification rate. The interaction between all our tested variables is most likely complex, and we would not expect to be able to perfectly reconstruct the precise impacts of each driver on diversification, as is reflected in our results. Nevertheless, it is clear that most of our statistically significant correlations show a clear transfer of information from one time series to another, which supports our interpretation that both environmental change and biotic competition played a role in driving speciation and extinction in Pseudosuchia. For marine lineages, speciation is most strongly associated with global temperature, whereas the closest association with extinction is competition. By contrast, speciation in terrestrial and freshwater lineages is most strongly linked to biotic competition, while extinction is most closely associated with sea level. Therefore, while we show that both biotic and abiotic drivers have shaped pseudosuchian macroevolution, their relative contributions differ, which is constrained by ecology.

In summary, we show that the diversification dynamics of Pseudosuchia have been shaped over macroevolutionary timescales by a complex interplay of biotic and abiotic factors, as well as ecology. These intrinsic biotic effects, often referred to as the ‘Red Queen’ hypothesis^6^, have been typically thought to operate at or within the species level and over geologically short timescales. By contrast, the effect of extrinsic changes in the physical environment, known as the ‘Court Jester’ hypothesis, is thought to operate over much longer timescales^6^. Recent research, however, shows an influence of biotic drivers at scales greater than 40 Myr^84^; therefore, the reality is undoubtedly more complex than previously characterized. Similar to the patterns observed in foraminifera^9^ and sharks^8,9^, we find that neither the Red Queen nor the Court Jester was the dominant force in shaping pseudosuchian diversity through time; rather, we find evidence for a pluralistic model in which their interaction varies across ecologies. This unexpected complexity is revealed by the decoupling of speciation and extinction rates, which can only be evaluated by taking into account past diversity and the fossil record.

## Conclusion

In view of the current focus on using drivers of diversification rates as predictors of clade responses to anthropogenically driven climate change, our findings show that fossils must be included in diversification analyses if we wish to make predictions about the drivers of both speciation and extinction in today’s at-risk clades. This is becoming increasingly important as the number of species threatened with extinction by climate change continues to rise and is particularly consequential for clades that have very low extant diversity such as today’s remaining pseudosuchians. Although many studies have explored drivers of diversification in a phylogenetic framework^30,85–92^, our study combines both extant and extinct taxa to explicitly model both speciation and extinction rates in a phylogenetic framework, allowing a more nuanced perspective of the drivers of diversification through time. Furthermore, this type of diversification study has not previously been carried out on such a temporally extensive group: similar studies do not yet exist for other vertebrate groups with comparable evolutionary histories. The fossil record provides a unique window onto the likely drivers that led to lineage success and decline, and that may ultimately lead to their extinction, and the inclusion of extinct taxa in diversification analyses is one way in which the potential of the fossil record can be leveraged.

## Methods

### Phylogenetic tree construction

The phylogeny was generated via a ‘metatree’ approach^93^. This approach is similar to formal supertree analysis but differs in that the input is not published trees but the original character matrices or sequence alignments that are themselves reanalysed to generate more complete sets of source topologies. Initially, we input all 1,594 available matrices classified as non-dinosaurian archosauromorphs obtained from an online resource^94^ (see Supplementary Information 1 for a full list). Only those matrices containing at least three pseudosuchian taxa were retained for additional analysis. From these matrices, the most parsimonious trees (MPTs) were generated until all unique bipartitions for a data set were sampled. Taxonomy was reconciled via the Paleobiology Database (https://paleobiodb.org/)^95^ to standardize nomenclature (for example, remove synonyms). These were then encoded into a matrix representation with parsimony matrix, using standard Baum and Ragan coding^96^. We also included a molecular tree containing 23 extant taxa^97^. This tree was upweighted in the final matrix to account for the disproportionate influence of morphology on the position of *Gavialis gangeticus*^97^. The resulting matrix contained 804 taxa (see Supplementary Data 1 for the final matrix representation with parsimony matrix). We analysed the matrix in TNT v1.5^98^ using the ‘xmult=10’ option and ran 1,000 replicates for the analysis. The analysis found 1,320 MPTs of length 1,100,897. The strict and majority rule consensus trees were very poorly resolved, and as the diversification analyses require fully resolved trees, we inferred a Maximum Agreement Subtree (MAST) in PAUP* 4.0a165^99^ to remove unstable taxa. Due to computational constraints, we computed the MAST from a random sample of 10% of the MPTs. After removal of unstable taxa via MAST construction, the final phylogeny contained 534 taxa and was fully resolved.

### Time calibration

Parsimony methods do not return trees with meaningful branch lengths; therefore, we used external fossil age data to time-calibrate the tree based on the Paleobiology Database and a review of the literature^15,17^. Age ranges were standardized to the Geological Society of America Time Scale v5.0^100^, with regional ages being converted to their equivalent age in the global timescale (see Supplementary Data 2 for fossil age data). We then used the fossilized birth–death tip dating method, implemented in BEAST2 v2.6.0^101^, to time-scale the phylogeny. Molecular data^97^ were used to calibrate the divergence times of extant lineages, whereas the stratigraphically oldest known occurrence of each fossil species, which is clearly attributable to that taxon using an autapomorphy-based approach, was used to calibrate divergence dates for extinct taxa. We set our phylogeny as a topological constraint and set uniform calibration priors based on the fossil occurrence dates. The root of the tree was set to a minimum age of 260 Ma, which is currently the oldest supported age for the origin of Pseudosuchia^21,102,103^. The proportion of living species sampled was set to 0.88 on the basis that *Crocodylus suchus*, *G. gangeticus, Mecistops leptorhynchus* and *Paleosuchus trigonatus* were not present in the final topology, whereas all other settings were set to the default. We ran the analysis for 10,000,000 generations, resulting in a posterior distribution of 9,001 phylogenies. We used TreeAnnotator, implemented in BEAST2^101^, to compute the maximum clade credibility tree for use in all downstream analyses. See Supplementary Data 3 for the BEAST2 input file.

### Diversification dynamics

Diversification dynamics were modelled from the phylogeny via Fossil BAMM v.2.6^104^, which is an extension of the BAMM Bayesian framework^105^ that uses a Markov Chain Monte Carlo approach to calculate diversification rates. It is explicitly designed to allow the calculation of both speciation and extinction rates, as well as net diversification rates, in phylogenies that contain extinct taxa. The number of fossil occurrences of taxa sampled in the phylogeny was set to 1,639, based on data in the Paleobiology Database and in recent publications^15,17^. Synonyms were removed from these data to establish the number of unique fossil operational taxonomic units, with this value then being combined with the number of extant species without fossil data to give the total number of known pseudosuchian taxa. From this, we calculated a global sampling fraction of 0.7. Four chains were executed for the analysis, each with a total of 30 million generations executed, with a minimum clade size of five taxa used to aid convergence. Ten thousand of the results were stored, with 10% discarded as ‘burn-in’, leaving 9,001 samples for subsequent analysis with regards to temperature correlation. For details of the Fossil BAMM set-up, see Supplementary Data 4.

To evaluate diversification dynamics with respect to environmental change, we first subdivided the tree into marine, freshwater and exclusively terrestrial taxa, based on previous compilations^15,24,38^, coupled with an exhaustive literature search (see Supplementary Data 2 for details). We used the R package BAMMtools^106^ to extract subtrees for each of these categories from the main supertree. We then used the speciation, extinction and net diversification rate curves obtained from Fossil BAMM^104^, extracted with BAMMtools^106^, to test for correlations against two global palaeotemperature^107,108^ and eustatic sea level^109^ time series (Supplementary Data 2). These data sets were chosen for two reasons: (1) the extent of geological time covered and (2) the relatively smooth nature of the time series. A more recently published sea level data set^110^ only extends back as far as 179 Ma, while other temperature data sets^111^ are not smooth enough to correlate against diversification rate data. Before carrying out the correlations, the temperature time series was smoothed using a Tukey running mean to remove noise. For all environmental time series, the values were linearly interpolated to the same time values available in the diversification rate data, which occurred in 0.1 Myr bins. We used all 9,001 simulations as modelled from the phylogeny, resulting in a distribution for each set of rates and environmental variables (Table 1). We used detrended cross-correlation analysis to account for non-stationarity and autocorrelation between time series^112^. To test for diversity dependence as an indicator of biotic interactions, we also correlated the number of LTT with speciation, extinction and net diversification rates. We estimated the LTT for each ecology using the R package ‘ape’ v.5.7-1^113^, then correlated the resulting time series against all 9,001 realizations of the speciation, extinction and net diversification curves. We then tested for significance using a Wilcoxon signed-rank test. As for the environmental analyses, we used detrended cross-correlation analysis to account for non-stationarity and autocorrelation between time series^112^. All analyses were carried out in R v.3.6.0^114^.

### Information transfer

Information theory has previously been used to test for causality in palaeontological and ecological data sets^115–117^. Transfer entropy is a directional method for measuring information that quantifies how temporal change in one time series informs that of another^118^. Transfer entropy is based on the mutual information method^118^ but takes into account the direction of information transfer by assuming that the processes can be described by a Markov model. It reduces to a linear Granger causality process, whereby a signal in one time series gives a linear response to the second time series. However, it makes fewer assumptions regarding the linearity of the processes involved and is therefore more suitable for analysing causality when the processes involved are unknown^119,120^, as is the case for information flow in natural systems. All our transfer entropy analyses were implemented in *R*^114^ using the package RTransferEntropy v.0.2.21^121^. Our data were placed into bins of equal length, and we allowed the number of bins to vary to minimize the number of bins containing either zero or single counts, as this can lead to bias in the results^115^. To determine the number of Markov states that best fit the system, we used a hidden Markov model, implemented in the R package ‘depmixS4’ v.1.5-0^122^, varying the number of states between 2 and 20. The number of states in the model with the lowest Akaike Information Criterion value was then used in the transfer entropy calculation. We then calculated transfer entropy 100 times for each pair of time series, that is, speciation and extinction rates for each habitat partition with each of our two abiotic variables and our one biotic variable. A higher positive value of transfer entropy indicates more information transfer. Statistical significance was calculated at the 95% confidence level, and only statistically significant results were retained (Table 1).

### Accounting for phylogenetic and temporal uncertainty

Both phylogenetic and temporal uncertainty can impact the results of large-scale macroevolutionary studies, and it has been questioned whether or not large, synthetic phylogenies accurately represent the underlying data and are therefore suitable for conducting macroevolutionary analyses^123^. Therefore, we carried out additional analyses to assess the impact of both phylogenetic and temporal variation on our results.

To assess phylogenetic uncertainty, we ran our analyses on two alternative topologies, one with Thalattosuchia as sister to Crocodyliformes and one with Phytosauria excluded from Pseudosuchia. These alternative topologies represent the most significant source of phylogenetic uncertainty within Pseudosuchia^124,125^. The impact of temporal variation on our results was achieved by taking a sample of 20 topologies from the posterior distribution, each of which were identical topologically but with differing node dates. This generated an additional 22 phylogenies in total (2 with an alternative topology and 20 with the same topology but differing node dates). Our full set of analyses was re-run for each of these 22 trees to assess whether, and to what extent, phylogenetic and temporal uncertainty impact upon our results.

### Reporting summary

Further information on research design is available in the Nature Portfolio Reporting Summary linked to this article.

### Supplementary information

Supplementary Information Supplementary Information 1—File containing a reference list for all source phylogenies included in the phylogeny.

Reporting Summary

Supplementary Data 1 Final MRP data matrix in TNT format.

Supplementary Data 2 Input file for time calibration.

Supplementary Data 3 Input file for diversification dynamics analysis.

Supplementary Data 4 Input data for the following: list of all fossil ages used to time-calibrate the phylogeny, habitat classifications used to partition the data set when carrying out correlation analyses, and all time series used in the correlation analyses. Includes proxies for palaeotemperature and eustatic sea level plus lineage through time data for each of the habitat partitions.

## Data Availability

The authors declare that all data supporting the findings of this study are available within the article and its supplementary information files.

## Code availability

For equity-related reasons custom code will be available on request to the corresponding author.
