# Supplementary material for: Decoupling speciation and extinction reveals both abiotic and biotic drivers shaped 250 million years of diversity in crocodile-line archosaurs
Source: Nat Ecol Evol. 2023 Dec 4;8(1):121–32. doi: 10.1038/s41559-023-02244-0 (PMC10781641; doi:10.1038/s41559-023-02244-0)
Supplement: Supplementary file 1 — Supplementary Information 1—File containing a reference list for all source phylogenies included in the phylogeny. [file 41559_2023_2244_MOESM1_ESM.pdf]

nature ecology & evolution

Article https://doi.org/10.1038/s41559-023-02244-0

# Decoupling speciation and extinction reveals both abiotic and biotic drivers shaped 250 million years of diversity in crocodile-line archosaurs

In the format provided by the
authors and unedited

## Supplementary Information 1: Source tree citations


### 2020

Johnson, M. M., Young, M. T. & Brusatte, S. L. 2020. Re-description of two contemporaneous mesorostrine teleosauroids (Crocodylomorpha: Thalattosuchia) from the Bathonian of England and insights into the early evolution of Machimosaurini. Zoological Journal of the Linnean Society 189, 449-482.

Rio, J. P., Mannion, P. D., Tschopp, E., Martin, J. E. & Delfino, M. 2020. Reappraisal of the morphology and phylogenetic relationships of the alligatoroid crocodylian *Diplocynodon hantoniensis* from the late Eocene of the United Kingdom. Zoological Journal of the Linnean Society 188, 579-629.

### 2019

Butler, R. J., Ezcurra, M. D., Liu, J., Sookias, R. B. & Sullivan, C. 2019. The anatomy and phylogenetic position of the erythrosuchid archosauriform *Guchengosuchus shiguaiensis* from the earliest Middle Triassic of China. PeerJ 7, e6435.

Butler, R. B., Jones, A. S., Buffetaut, E., Mandl, G. W., Scheyer, T. M. & Schultz, O. 2019. Description and phylogenetic placement of a new marine species of phytosaur (Archosauriformes: Phytosauria) from the Late Triassic of Austria. Zoological Journal of the Linnean Society 1, 198-228.

Ezcurra, M. D., Gower, D. J., Sennikov, A. G. & Butler, R. J. 2019. The osteology of the holotype of the early erythrosuchid *Garjainia prima* (Diapsida: Archosauromorpha) from the upper Lower Triassic of European Russia. Zoological Journal of the Linnean Society 185, 717-783.

Foffa, D., Johnson, M. M., Young, M. T., Steel, L. & Brusatte, S. L. 2019. Revision of the Late Jurassic deep-water teleosauroid crocodylomorph *Teleosaurus megarhinus* Hulke, 1871 and evidence of pelagic adaptations in Teleosauroidea. PeerJ 7, e6646.

Geroto, C. F. C. & Bertini, R. J. 2019. New material *of Pepesuchus* (Crocodyliformes; Mesoeucrocodylia) from the Bauru Group: implications about its phylogeny and the age of the Adamantina Formation. Zoological Journal of the Linnean Society 185, 312-334.

Holgado, B., Pegas, R. V., Canudo, J. I., Fortuny, J., Rodrigues, T., Company, J. & Kellner, A. W. A. 2019. On a new crested pterodactyloid from the Early Cretaceous of the Iberian Peninsula and the radiation of the clade Anhangueria. Scientific Reports 9, 4940.

Jouve, S., Sarigul, V., Steyer, J.-S. & Sen, S. 2019. The first crocodylomorph from the Mesozoic of Turkey (Barremian of Zonguldak) and the dispersal of the eusuchians during the Cretaceous. Journal of Systematic Palaeontology 17, 111-128.

Noto, C. R., Drumheller, S. K., Adams, T. L. & Turner, A. H. 2019.. An enigmatic small neosuchian crocodyliform from the Woodbine Formation of Texas. The Anatomical Record 303, 801-812.

Wilberg, E. W., Turner, A. H. & Brochu, C. A. 2019. Evolutionary structure and timing of major habitat shifts in Crocodylomorpha. Scientific Reports 9, 514.

### 2018

Baron, M. G. & Williams, M. E. 2018. A re-evaluation of the enigmatic dinosauriform *Caseosaurus crosbyensis* from the Late Triassic of Texas, USA and its implications for early dinosaur evolution. Acta Palaeontologica Polonica 63, 129-145.

Barrios, F., Bona, P., Paulina-Carabajal, A. & Gasparini, Z. 2018. Re-description of the cranio-mandibular anatomy of *Notosuchus terrestris* (Crocodyliformes, Mesoeucrocodylia) from the Upper Cretaceous of Patagonia. Cretaceous Research 83, 3-39.

Britt, B. B., Dalla Vecchia, F. M., Chure, D. J., Engelmann, G. F., Whiting, M. F. & Scheetz, R. D. 2018. *Caelestiventus hanseni gen. et sp. nov.* extends the desert-dwelling pterosaur record back 65 million years. Nature Ecology & Evolution 2, 1386-1392.

De Oliveira, T. M., Oliveira, D., Schultz, C. L., Kerber, L. & Pinheiro, F. L. 2018. Tanystropheid archosauromorphs in the Lower Triassic of Gondwana. Acta Palaeontologica Polonica 63, 713-723.

Dollman, K. N., Clark, J. M., Norell, M. A., Xu, X. & Choiniere, J. N. 2018. Convergent evolution of a eusuchian-type secondary palate within Shartegosuchidae. American Museum Novitates 3901, 1-23.

Ezcurra, M. D. v Butler, R. J. 2018. The rise of the ruling reptiles and ecosystem recovery from the Permo-Triassic mass extinction. Proceedings of the Royal Society B: Biological Sciences 285, 20180361.

Foffa, D., Young, M. T., Brusatte, S. L., Graham, M. R. & Steel, L. 2018. A new metriorhynchid crocodylomorph from the Oxford Clay Formation (Middle Jurassic) of England, with implications for the origin and diversification of Geosaurini. Journal of Systematic Palaeontology 16, 1123-1143.

Gentil, A. R. & Ezcurra, M. D. 2018. Reconstruction of the masticatory apparatus of the holotype of the rhynchosaur *Hyperodapedon sanjuanensis* from the Late Triassic of Argentina: implications for the diagnosis of the species. Ameghiniana 55, 137-149.

Hoffman, D. K., Heckert, A. B. & Zanno, L. E. 2018. Under the armor: x-ray computed tomographic reconstruction of the internal skeleton of *Coahomasuchus chathamensis* (Archosauria: Aetosauria) from the Upper Triassic of North Carolina, USA, and a phylogenetic analysis of Aetosauria. PeerJ 6, e4368.

Iori, F. V., Marinho, T. S., Carvalho, I. S. & Frare, L. A. S. 2018. Cranial morphology of *Morrinhosuchus luziae* (Crocodyliformes, Notosuchia) from the Upper Cretaceous of the Bauru Basin, Brazil. Cretaceous Research 86 41-52.

Jones, A. S. & Butler, R. J., 2018. A new phylogenetic analysis of Phytosauria (Archosauria: Pseudosuchia) with the application of continuous and geometric morphometric character coding. PeerJ 6, e5901.

Kubo, T., Shibata, M., Naksri, W., Jintasakul, P. & Azuma, Y. 2018. The earliest record of Asian Eusuchia from the Lower Cretaceous Khok Kruat Formation of northeastern Thailand. Cretaceous Research 82, 21-28.

Lacerda, M. B., Franca, M. A. G. & Schultz, C. L. 2018. A new erpetosuchid (Pseudosuchia, Archosauria) from the Middle-Late Triassic of Southern Brazil. Zoological Journal of the Linnean Society 184, 804-824.

Leal, M. E. C., Pegas, R. V., Bonde, N. & Kellner, A. W. A. 2018. Cervical vertebrae of an enigmatic pterosaur from the Crato Formation (Lower Cretaceous, Araripe Basin, NE Brazil). Geological Society of London Special Publications 455, 195-208.

Leardi, J. M., Pol, D. & Gasparini, Z. 2018. New Patagonian baurusuchids (Crocodylomorpha; Notosuchia) from the Bajo de la Carpa Formation (Upper Cretaceous;

Neuquen, Argentina): new evidences of the early sebecosuchian diversification in Gondwana. Comptes Rendus Palevol 17, 504-521.

Lee, M. S. Y. & Yates, A. M. 2018. Tip-dating and homoplasy: reconciling the shallow molecular divergences of modern gharials with their long fossil record. Proceedings of the Royal Society B: Biological Sciences 285, 20181071.

Lio, G., Agnolin, F. L., Martinelli, A. G., Ezcurra, M. D. & Novas, F. E. 2018. New specimen of the enigmatic, Late Cretaceous crocodyliform *Neuquensuchus universitas* sheds light on the anatomy of the species. Cretaceous Research 83, 62-74.

Longrich, N. R., Martill, D. M. & Andres, B. 2018. Late Maastrichtian pterosaurs from North Africa and mass extinction of Pterosauria at the Cretaceous-Paleogene boundary. PLOS Biology 16, e2001663.

Lu, J.-C., Meng, Q.-J., Wang, B.-P., Liu, D., Shen, C.-Z. & Zhang, Y.-G. 2018. Short note on a new anurognathid pterosaur with evidence of perching behaviour from Jianchang of Liaoning Province, China. Geological Society of London Special Publications 455, SP455.

Martin, J. E., Suteethorn, S., Lauprasert, K., Tong, H.-Y., Buffetaut, E., Liard, R., Salaviale, C., Deesri, U., Suteethorn, V. & Claude, J., 2018. A new freshwater teleosaurid from the Jurassic of northeastern Thailand. Journal of Vertebrate Paleontology 38, e1549059.

Muller, R. T., Langer, M. C. & Dias-da-Silva, S. 2018. Ingroup relationships of Lagerpetidae (Avemetatarsalia: Dinosauromorpha): a further phylogenetic investigation on the understanding of dinosaur relatives. Zootaxa 4392, 149-158.

Osi, A., Young, M. T., Galacz, A. & Rabi, M. 2018. A new large-bodied thalattosuchian crocodyliform from the Lower Jurassic (Toarcian) of Hungary, with further evidence of the mosaic acquisition of marine adaptations in Metriorhynchoidea. PeerJ 6, e4668.

Parker, W. G. 2018. Anatomical notes and discussion of the first described aetosaur *Stagonolepis robertsoni* (Archosauria: Suchia) from the Upper Triassic of Europe, and the use of plesiomorphies in aetosaur biochronology. PeerJ 6, e5455.

Parker, W. G. 2018. Redescription *of Calyptosuchus* (*Stagonolepis*) *wellesi* (Archosauria: Pseudosuchia: Aetosauria) from the Late Triassic of the Southwestern United States with a discussion of genera in vertebrate paleontology. PeerJ 6, e4291.

Peecook, B. R., Smith, R. M. H. & Sidor, C. A. 2018. A novel archosauromorph from Antarctica and an updated review of a high-latitude vertebrate assemblage in the wake of the end-Permian mass extinction. Journal of Vertebrate Paleontology 38, e1536664.

Pegas, R. V., Costa, F. R. & Kellner, A. W. A. 2018. New information on the osteology and a taxonomic revision of the *genus Thalassodromeus* (Pterodactyloidea, Tapejaridae, Thalassodrominae). Journal of Vertebrate Paleontology 38, e1443273.

Pritchard, A. C., Gauthier, J. A., Hanson, M., Bever, G. S. & Bhullar, B.-A. S. 2018. A tiny Triassic saurian from Connecticut and the early evolution of the diapsid feeding apparatus. Nature Communications 9, 1213.

Ristevski, J., Young, M. T., de Andrade, M. B. & Hastings, A. K. 2018. A new species of *Anteophthalmosuchus* (Crocodylomorpha, Goniopholididae) from the Lower Cretaceous of the Isle of Wight, United Kingdom, and a review of the genus. Cretaceous Research 84, 340-383.

Sarigul, V., Agnolin, F. & Chatterjee, S. 2018. Description of a multitaxic bone assemblage from the Upper Triassic Post Quarry of Texas (Dockum Group), including a new small basal dinosauriform taxon. Historia Natural Tercera Serie 8, 5-24.

Spiekman, S. N. F. 2018. A new specimen of *Prolacerta broomi* from the lower Fremouw Formation (Early Triassic) of Antarctica, its biogeographical implications and a taxonomic revision. Scientific Reports 8, 17996.

Vidovic, S. U. & Martill, D. M. 2018. The taxonomy and phylogeny of *Diopecephalus kochi* (Wagner, 1837) and '*Germanodactylus rhamphastinus*' (Wagner, 1851). Geological Society of London Special Publications SP455.12.

Wu, X.-C., Li, C. & Wang, Y.-Y. 2018. Taxonomic reassessment and phylogenetic test of *Asiatosuchus nanlingensis* Young, 1964 and *Eoalligator chunyii* Young, 1964. Vertebrata PalAsiatica, 56 137-146.

**2017**

Adams, T. L., Noto, C. R. & Drumheller, S. 2017. A large neosuchian crocodyliform from the Upper Cretaceous (Cenomanian) Woodbine Formation of North Texas. Journal of Vertebrate Paleontology 37, e1349776.

Buscalioni, A. D. 2017. The Gobiosuchidae in the early evolution of Crocodyliformes. Journal of Vertebrate Paleontology 37, e1324459.

Butler, R. J., Nesbitt, S. J., Charig, A. J., Gower, D. J. & Barrett, P. M. 2017. *Mandasuchus tanyauchen*, *gen. et sp. nov.*, a pseudosuchian archosaur from the Manda Beds (?Middle Triassic) of Tanzania. Journal of Vertebrate Paleontology 37, 96-121.

Cidade, G. M., Solorzano, A., Rincon, A. D., Riff, D. & Hsiou, A. S. 2017. A *new Mourasuchus* (Alligatoroidea, Caimaninae) from the late Miocene of Venezuela, the phylogeny of Caimaninae and considerations on the feeding habits of *Mourasuchus*. PeerJ 5, e3056.

Dal Sasso, C., Pasini, G., Fleury, G. & Maganuco, S. 2017. *Razanandrongobe sakalavae*, a gigantic mesoeucrocodylian from the Middle Jurassic of Madagascar, is the oldest known notosuchian. PeerJ 5, e3481.

Ezcurra, M. D., Fiorelli, L. E., Martinelli, A. G., Rocher, S., von Baczko, M. B., Ezpeleta, M., Taborda, J. R. A., Hechenleitner, E. M., Trotteyn, M. J. & Desojo, J. B. 2017. Deep faunistic turnovers preceded the rise of dinosaurs in southwestern Pangaea. Nature Ecology & Evolution 1, 1477-1483.

Langer, M. C., da Rosa, A. A. S. & Montefeltro, F. C. 2017. *Supradapedon revisited*: geological explorations in the Triassic of southern Tanzania. PeerJ 5, e4038.

Leardi, J. M., Pol, D. & Clark, J. M. 2017. Detailed anatomy of the braincase of *Macelognathus vagans* Marsh, 1884 (Archosauria, Crocodylomorpha) using high resolution tomography and new insights on basal crocodylomorph phylogeny. PeerJ 5, e2801.

Lecuona, A., Desojo, J. B. & Pol, D. 2017. New information on the postcranial skeleton of *Gracilisuchus stipanicicorum* (Archosauria: Suchia) and reappraisal of its phylogenetic position. Zoological Journal of the Linnean Society 181, 638-677.

Liu, J., Organ, C. L., Benton, M. J., Brandley, M. C. & Aitchison, J. C. 2017. Live birth in an archosauromorph reptile. Nature Communications 8, 14445.

Muller, R. T. 2017. Are the dinosauromorph femora from the Upper Triassic of Hayden Quarry (New Mexico) three stages in a growth series of a single taxon? Anais da Academia Brasileira de Ciencias, 89 835-839.

Nesbitt, S. J., Butler, R. J., Ezcurra, M. D., Charig, A. J. & Barrett, P. M. 2017. The anatomy of *Teleocrater rhadinus*, an early avemetatarsalian from the lower portion of the Lifua Member of the Manda Beds (Middle Triassic). Journal of Vertebrate Paleontology 37, 142-177.

Nesbitt, S. J., Butler, R. J., Ezcurra, M. D., Barrett, P. M., Stocker, M. R., Angielczyk, K. D., Smith, R. M. H., Sidor, C. A., niedzwiedzki, G., Sennikov, A. G. & Charig, A. J. 2017. The earliest bird-line archosaurs and the assembly of the dinosaur body plan. Nature 544, 484-487.

Schwarz, D., Raddatz, M. & Wings, O. 2017. *Knoetschkesuchus langenbergensis gen. nov. sp. nov.*, a new atoposaurid crocodyliform from the Upper Jurassic Langenberg Quarry (Lower Saxony, northwestern Germany), and its relationships to Theriosuchus. PLOS ONE 12, e0160617.

Sengupta, S., Ezcurra, M. D. & Bandyopadhyay, S. 2017. A new horned and long-necked herbivorous stem-archosaur from the Middle Triassic of India. Scientific Reports 7, 8366.

Stocker, M. R., Zhao, L.-J., Nesbitt, S. J., Wu, X.-C. & Li, C. 2017. A short-snouted, Middle Triassic phytosaur and its implications for the morphological evolution and biogeography of Phytosauria. Scientific Reports 7, 46028.

Unwin, D. M. & Martill, D. M. 2017. Systematic reassessment of the first Jurassic pterosaur from Thailand. Geological Society of London Special Publications 455, SP455.13.

Wang, X.-L., Jiang, S.-X., Zhang, J.-Q., Cheng, X., Yu, X.-F., Li, Y.-M., Wei, G.-J. & Wang, X.-L. 2017. New evidence from China for the nature of the pterosaur evolutionary transition. Scientific Reports 7, 42763.

Wilberg, E. W. 2017. Investigating patterns of crocodyliform cranial disparity through the Mesozoic and Cenozoic. Zoological Journal of the Linnean Society 181, 189-208.

Wu, W.-H., Zhou, C.-F. & Andres, B. 2017. The toothless pterosaur J*idapterus edentus* (Pterodactyloidea: Azhdarchoidea) from the Early Cretaceous Jehol Biota and its paleoecological implications. PLOS ONE 12, e0185486.

Young, M. T., Hastings, A. K., Allain, R. & Smith, T. J. 2017. Revision of the enigmatic crocodyliform *Elosuchus felixi* de Lapparent de Broin, 2002 from the Lower-Upper Cretaceous boundary of Niger: potential evidence for an early origin of the clade Dyrosauridae. Zoological Journal of the Linnean Society 179, 377-403.

Zhou, C.-F., Gao, K.-Q., Yi, H.-Y., Xue, J.-Z., Li, Q.-G. & Fox, R. C. 2017. Earliest filter-feeding pterosaur from the Jurassic of China and ecological evolution of Pterodactyloidea. Royal Society Open Science 4, 160672.

**2016**

Cheng, X., Jiang, S.-X., Wang, X.-L. & Kellner, A. W. A. 2016. New information on the Wukongopteridae (Pterosauria) revealed by a new specimen from the Jurassic of China. PeerJ 4, e2177.

Codorniu, L., Carabajal, A. P., Pol, D., Unwin, D. & Rauhut, O. W. M. 2016. A Jurassic pterosaur from Patagonia and the origin of the pterodactyloid neurocranium. PeerJ 4, e2311.

Drymala, S. M. & Zanno, L. E. 2016. Osteology of *Carnufex carolinensis* (Archosauria: Psuedosuchia) from the Pekin Formation of North Carolina and its implications for early crocodylomorph evolution. PLOS ONE 11, e0157528.

Ezcurra, M. D. 2016. The phylogenetic relationships of basal archosauromorphs, with an emphasis on the systematics of proterosuchian archosauriforms. PeerJ 4, e1778.

Ezcurra, M. D., Montefeltro, F. & Butler, R. J. 2016. The early evolution of rhynchosaurs. Frontiers in Ecology and Evolution 3, 142.

Fanti, F., Miyashita, T., Cantelli, L., Mnasri, F., Dridi, J., Contessi, M. & Cau, A. 2016. The largest thalattosuchian (Crocodylomorpha) supports teleosaurid survival across the Jurassic-Cretaceous boundary. Cretaceous Research 61, 263-274.

Fiorelli, L. E., Leardi, J. M., Hechenleitner, E. M., Pol, D., Basilici, G. & Grellet-Tinner, G. 2016. A new Late Cretaceous crocodyliform from the western margin of Gondwana (La Rioja Province, Argentina). Cretaceous Research 60, 194-209.

Godoy, P. L., Bronzati, M., Eltink, E., Marsola, J. C. A., Cidade, G. M., Langer, M. C. & Montefeltro, F. C. 2016. Postcranial anatomy of Pissarrachampsa sera (Crocodyliformes, Baurusuchidae) from the Late Cretaceous of Brazil: insights on lifestyle and phylogenetic significance. PeerJ 4, e2075.

Kammerer, C. F., Butler, R. J., Bandyopadhyay, S. & Stocker, M. R. 2016. Relationships of the Indian phytosaur *Parasuchus hislopi* Lydekker, 1885. Papers in Palaeontology 2, 1-23.

Lacerda, M. B., Mastrantonio, B. M., Fortier, D. C. & Schultz, C. L. 2016. New insights on *Prestosuchus chiniquensis* Huene, 1942 (Pseudosuchia, Loricata) based on new specimens from the "Tree Sanga" Outcrop, Chiniqua Region, Rio Grande do Sul, Brazil. PeerJ 4, e1622.

Lessner, E. J., Stocker, M. R., Smith, N. D., Turner, A. H., Irmis, R. B. & Nesbitt, S. J. 2016. A new rauisuchid (Archosauria, Pseudosuchia) from the Upper Triassic (Norian) of New Mexico increases the diversity and temporal range of the clade. PeerJ 4, e2336.

Li, C., Wu, X.-C., Zhao, L.-J., Nesbitt, S. J., Stocker, M. R. & Wang, L.-T. 2016. A new armored archosauriform (Diapsida: Archosauromorpha) from the marine Middle Triassic of China, with implications for the diverse life styles of archosauriforms prior to the diversification of Archosauria. The Science Of Nature 103, 95.

Li, Z.-G., Jiang, D.-Y., Rieppel, O., Motani, R., Tintori, A., Sun, Z.-Y. & Ji, C. 2016. A new species *of Xinpusaurus* (Reptilia, Thalattosauria) from the Ladinian (Middle Triassic) of Xingyi, Guizhou, southwestern China. Journal of Vertebrate Paleontology 36, e1218340.

Lu, J.-C., Kundrat, M. & Shen, C. 2016. New material of the *pterosaur Gladocephaloideus Lu* et al., 2012 from the Early Cretaceous of Liaoning Province, China, with comments on its systematic position. PLOS ONE 11, e0154888.

Martin, J. E. & de Broin, F. L. 2016. A miniature notosuchian with multicuspid teeth from the Cretaceous of Morocco. Journal of Vertebrate Paleontology 36, e1211534.

Martin, J. E., Delfino, M., Garcia, G., Godefroit, P., Berton, S. & Valentin, X. 2016. New specimens of *Allodaposuchus precedens* from France: intraspecific variability and the diversity of European Late Cretaceous eusuchians. Zoological Journal of the Linnean Society 176, 607-631.

Narvaez, I., Brochu, C. A., Escaso, F., Perez-Garcia, A. & Ortega, F. 2016. New Spanish Late Cretaceous eusuchian reveals the synchronic and sympatric presence of two allodaposuchids. Cretaceous Research 65, 112-125.

Niedzwiedzki, G., Sennikov, A. & Brusatte, S. L. 2016. The osteology and systematic position of *Dongusuchus efremovi* Sennikov, 1988 from the Anisian (Middle Triassic) of Russia. Historical Biology 28, 550-570.

Parker, W. G. 2016. Revised phylogenetic analysis of the Aetosauria (Archosauria: Pseudosuchia); assessing the effects of incongruent morphological character sets. PeerJ 4, e1583.

Pegas, R. V., Leal, M. E. C., Wilhelm, A. & Kellner, A. 2016. A basal tapejarine (Pterosauria; Pterodactyloidea; Tapejaridae) from the Crato Formation, Early Cretaceous of Brazil. PLOS ONE 11, e0162692.

Pinheiro, F. L., Franca, M. A. G., Lacerda, M. B., Butler, R. J. & Schultz, C. L. 2016. An exceptional fossil skull from South America and the origins of the archosauriform radiation. Scientific Reports 6, 22817.

Roberto-da-Silva, L., Franca, M. A. G., Cabreira, S. F., Muller, R. T. & Dias-da-Silva, S. 2016. On the presence of the subnarial foramen in *Prestosuchus chiniquensis* (Pseudosuchia: Loricata) with remarks on its phylogenetic distribution. Anais da Academia Brasileira de Ciencias 88, 1309-1323.

Salas-Gismondi, R., Flynn, J. J., Baby, P., Tejada-Lara, J. V., Claude, J. & Antoine, P.-O. 2016. A new 13 million year old gavialoid crocodylian from proto-Amazonian mega-wetlands reveals parallel evolutionary trends in skull shape linked to longirostry. PLOS ONE 11, E0152453.

Schoch, R. R. & Desojo, J. B. 2016. Cranial anatomy of the aetosaur *Paratypothorax andressorum* Long & BaLLew, 1985, from the Upper Triassic of Germany and its bearing on aetosaur phylogeny. Neues Jahrbuch fur Geologie und Palaontologie Abhandlungen 279, 73-95.

Schultz, C. L., Langer, M. C. & Montefeltro, F. C. 2016. A new rhynchosaur from south Brazil (Santa Maria Formation) and rhynchosaur diversity patterns across the Middle-Late Triassic boundary. Palaontologische Zeitschrift 90, 593-609.

Sookias, R. B. 2016. The relationships of the Euparkeriidae and the rise of Archosauria. Royal Society Open Science 3, 150674.

Stocker, M. R., Nesbitt, S. J., Criswell, K. E., Parker, W. G., Witmer, L. M., Rowe, T. B., Ridgely, R. & Brown, M. A. 2016. A dome-headed stem archosaur exemplifies convergence among dinosaurs and their distant relatives. Current Biology 26 887-889.

Wang, Y.-Y., Sullivan, C. & Liu, J. 2016. Taxonomic revision of *Eoalligator* (Crocodylia, Brevirostres) and the paleogeographic origins of the Chinese alligatoroids. PeerJ 4, e2356.

**2015**

Bittencourt, J. S., Arcucci, A. B., Marsicano, C. A. & Langer, M. C. 2015. Osteology of the Middle Triassic archosaur *Lewisuchus admixtus* Romer (Chanares Formation, Argentina), its inclusivity, and relationships amongst early dinosauromorphs. Journal of Systematic Palaeontology 13, 189-219.

Bronzati, M., Montefeltro, F. C. & Langer, M. C. 2015. Diversification events and the effects of mass extinctions on Crocodyliformes evolutionary history. Royal Society Open Science 2, 140385.

Butler, R. J., Ezcurra, M. D., Montefeltro, F. C., Samathi, A. & Sobral, G. 2015. A new species of basal rhynchosaur (Diapsida: Archosauromorpha) from the early Middle Triassic of South Africa, and the early evolution of Rhynchosauria. Zoological Journal of the Linnean Society 174, 571-588.

Cleland, T. P., Schroeter, E. R., Zamdborg, L., Zheng, W.-X., Lee, J. E., Tran, J. C., Bern, M., Duncan, M. B., Lebleu, V. S., Ahlf, D. R., Thomas, P. M., Kalluri, R., Kelleher, N. L. & Schweitzer, M. H. 2015. Mass spectrometry and antibody-based characterization of blood vessels from *Brachylophosaurus canadensis*. Journal of Proteome Research 14, 5252-5262.

Ezcurra, M. D., Desojo, J. B. & Rauhut, O. W. M. 2015. Redescription and phylogenetic relationships of the proterochampsid *Rhadinosuchus gracilis* (Diapsida: Archosauriformes) from the early Late Triassic of southern Brazil. Ameghiniana 52, 391-417.

Halliday, T. J. D., Brandalise De Andrade, M., Benton, M. J. & Efrimov, M. B. 2015. A re-evaluation of goniopholidid crocodylomorph material from Central Asia: biogeographic and phylogenetic implications. Acta Palaeontologica Polonica 60, 291-312.

Hastings, A. K., Bloch, J. I. & Jaramillo, C. A. 2015. A new blunt-snouted dyrosaurid, *Anthracosuchus balrogus gen. et sp. nov.* (Crocodylomorpha, Mesoeucrocodylia), from the Palaeocene of Colombia. Historical Biology 27, 998-1020.

Headden, J. A. & Campos, H. B. N. 2015. An unusual edentulous pterosaur from the Early Cretaceous Romualdo Formation of Brazil. Historical Biology 27, 815-826.

Heckert, A. B., Schneider, V. P., Fraser, N. C. & Webb, R. A. 2015. A new aetosaur (Archosauria, Suchia) from the Upper Triassic Pekin Formation, Deep River Basin, North Carolina, U.S.A., and its implications for early aetosaur evolution. Journal of Vertebrate Paleontology 35, e881831.

Herrera, Y., Gasparini, Z. & Fernandez, M. S. 2015. Purranisaurus potens Rusconi, an enigmatic metriorhynchid from the Late Jurassic-Early Cretaceous of the Neuquen Basin. Journal of Vertebrate Paleontology 35, e904790.

Jouve, S., Bouya, B., Amaghzaz, M. & Meslouh, S. 2015. Maroccosuchus zennaroi (Crocodylia: Tomistominae) from the Eocene of Morocco: phylogenetic and palaeobiogeographical implications of the basalmost tomistomine. Journal of Systematic Palaeontology 13, 421-445.

Lautenschlager, S. & Rauhut, O. W. M. 2015. Osteology of *Rauisuchus tiradentes* from the Late Triassic (Carnian) Santa Maria Formation of Brazil, and its implications for rauisuchid anatomy and phylogeny. Zoological Journal of the Linnean Society 173, 55-91.

Leardi, J. M., Pol, D., Novas, F. E. & Suarez, M. 2015. The postcranial anatomy of *Yacarerani boliviensis* and the phylogenetic significance of the notosuchian postcranial skeleton. Journal of Vertebrate Paleontology 35, e995187.

Leardi, J. M., Fiorelli, L. E. & Gasparini, Z. 2015. Redescription and reevaluation of the taxonomical status of *Microsuchus schilleri* (Crocodyliformes: Mesoeucrocodylia) from the Upper Cretaceous of Neuquen, Argentina. Cretaceous Research 52, 153-166.

Liu, D.-X., Zhou, C.-F., Wang, J.-Q., Li, W.-G. & Wei, Q.-W. 2015. New data on the cervical morphology of the Chinese tapejarine.Historical Biology 27, 638-645.

Lu, J.-C., Pu, H.-Y., Wei, X.-F., Chang, H.-L. & Kundrat, M. 2015. A new rhamphorhynchid pterosaur (Pterosauria) from Jurassic deposits of Liaoning Province, China. Zootaxa 3911, 119-129.

Myers, T. S. 2015. First North American occurrence of the toothed pteranodontoid pterosaur Cimoliopterus. Journal of Vertebrate Paleontology 35, e1014904.

Narvaez, I., Brochu, C. A., Escaso, F., Perez-Garcia, A. & Ortega, F. 2015. New crocodyliforms from southwestern Europe and definition of a diverse clade of European Late Cretaceous basal eusuchians. PLOS One 10, e0140679.

Nesbitt, S. J., Flynn, J. J., Pritchard, A. C., Parrish, J. M., Ranivoharimanana, L. & Wyss, A. R. 2015. Postcranial osteology of *Azendhosaurus madagaskarensis* (?Middle to Upper Triassic, Isalo Group, Madagascar) and its systematic position among stem archosaur reptiles. Bulletin of the American Museum of Natural History 398, 1-126.

Parrilla-Bel, J. & Canudo, J. I. 2015. Postcranial elements of Maledictosuchus riclaensis (Thalattosuchia) from the Middle Jurassic of Spain. Journal of Iberian Geology 41, 31-40.

Pritchard, A. C., Turner, A. H., Nesbitt, S. J., Irmis, R. B. & Smith, N. D. 2015. Late Triassic tanystropheids (Reptilia, Archosauromorpha) from northern New Mexico (Petrified Forest Member, Chinle Formation) and the biogeography, functional morphology, and evolution of Tanystropheidae. Journal of Vertebrate Paleontology 35, e911186.

Puertolas-Pascual, E., Canudo, J. I. & Sender, L. M. 2015. New material from a huge specimen of *Anteophthalmosuchus cf. escuchae* (Goniopholididae) from the Albian of Andorra (Teruel, Spain): Phylogenetic implications. Journal of Iberian Geology 41, 41-56.

Rabi, M. & Sebok, N. 2015. A revised Eurogondwana model: Late Cretaceous notosuchian crocodyliforms and other vertebrate taxa suggest the retention of episodic faunal links between Europe and Gondwana during most of the Cretaceous. Gondwana Research 28, 1197-1211.

Salas-Gismondi, R., Flynn, J. J., Baby, P., Tejada-Lara, J. V., Wesselingh, F. P. & Antoine, P.-O. 2015. A Miocene hyperdiverse crocodylian community reveals peculiar trophic dynamics in proto-Amazonian mega-wetlands. Proceedings of the Royal Society of London B 282, 20142490.

Sweetman, S. C., Pedreira-Segade, U. & Vidovic, S. U. 2015. A new bernissartiid crocodyliform from the Lower Cretaceous Wessex Formation (Wealden Group, Barremian) of the Isle of Wight, southern England. Acta Palaeontologica Polonica 60, 257-268.

Turner, A. H. 2015. A review of *Shamosuchus* and *Paralligator* (Crocodyliformes, Neosuchia) from the Cretaceous of Asia. PLOS ONE 10, e0118116.

Wilberg, E. W. 2015. A new metriorhynchoid (Crocodylomorpha, Thalattosuchia) from the Middle Jurassic of Oregon and the evolutionary timing of marine adaptations in thalattosuchian crocodylomorphs. Journal of Vertebrate Paleontology 35, e902846.

Wilberg, E. W. 2015. What's in an outgroup? The impact of outgroup choice on the phylogenetic position of Thalattosuchia (Crocodylomorpha) and the origin of Crocodyliformes. Systematic Biology 64, 621-637.

Wu, X.-C. & Brinkman, D. B. 2015. A new crocodylian (Eusuchia) from the uppermost Cretaceous of Alberta, Canada. Canadian Journal of Earth Sciences 52, 590-607.

Zanno, L. E., Drymala, S., Nesbitt, S. J. & Schneider, V. P. 2015. Early crocodylomorph increases top tier predator diversity during rise of dinosaurs. Scientific Reports 5, 9276.

2014

Adams, T. L. 2014. Small crocodyliform from the Lower Cretaceous (late Aptian) of central Texas and its systematic relationship to the evolution of Eusuchia. Journal of Paleontology 88, 1031-1049.

Aires, A. S. S., Kellner, A. W. A., Muller, R. T., Da Silva, L. R., Pacheco, C. P. & Dias-Da-Silva, S. 2014. New postcranial elements of the Thalassodrominae (Pterodactyloidea, Tapejariae) from the Romualdo Formation (Aptian-Albian), Santana Group, Araripe Basin, Brazil. Palaeontology 57, 343-355.

Andres, B., Clark, J. & Xu, X. 2014. The earliest pterodactyloid and the origin of the rogup. Current Biology 24, 1-6.

Bantim, R. A. M., Saraiva, A. A. F., Oliveira, G. R. & Sayao, J. M. 2014. A new toothed pterosaur (Pterodactyloidea: Anhangueridae) from the Early Cretaceous Romualdo Formation, NE Brazil. Zootaxa 3869, 201-223.

Blanco, A., Puertolas-Pascual, E., Marmi, J., Vila, B. & Selles, A. G. 2014. *Allodaposuchus palustris sp. nov.* from the Upper Cretaceous of Fumanya (southeastern Pyrenees, Iberian Peninsula): systematics, palaeoecology and palaeobiogeography of the enigmatic allodaposuchian crocodylians. PLOS ONE 9, e115837.

Brochu, C. A. & Jimenez-Vazquez, O. 2014. Enigmatic crocodyliforms from the early Miocene of Cuba. Journal of Vertebrate Paleontology 34, 1094-1101.

Butler, R. J., Rauhut, O. W. M., Stocker, M. R. & Bronowicz, R. 2014. Redescription of the phytosaurs *Paleorhinus* (‘*Francosuchus*’) *angustifrons* and *Ebrachosuchus neukami* from Germany, with implications for Late Triassic biochronology. Zoological Journal of the Linnean Society 170, 155-208.

Butler, R. J., Sullivan, C., Ezcurra, M. D., Liu, J., Lecuona, A. & Sookias, R. B. 2014. New clade of enigmatic early archosaurs yields insights into early pseudosuchian phylogeny and the biogeography of the archosaur radiation. BMC Evolutionary Biology 14, 128.

Cau, A. 2014. The affinities of '*Steneosaurus barettoni*' (Crocodylomorpha, Thalattosuchia), from the Jurassic of Northern Italy, and implications for cranial evolution among geosaurine metriorhynchids. Historical Biology 26, 433-440.

Farke, A. A., Henn, M. H., Woodward, S. J. & Xu, H. A. 2014. *Leidyosuchus* (Crocodylia: Alligatoroidea) from the Upper Cretaceous Kaiparowits Formation (late Campanian) of Utah, USA. PaleoBios 30, 72-88.

Fortier, D. C., De Souza-Filho, J. P., Guilherme, E., Maciente, A. A. R. & Schultz, C. L. 2014. A new specimen of *Caiman brevirostris* (Crocodylia, Alligatoridae) from the late Miocene of Brazil. Journal of Vertebrate Paleontology 34, 820-834.

Godoy, P. L., Montefeltro, F. C., Norell, M. A. & Langer, M. C. 2014. An additional baurusuchid from the Cretaceous of Brazil with evidence of interspecific predation among Crocodyliformes. PLOS ONE 9, e97138.

Gold, M. E. L., Brochu, C. A. & Norell, M. A. 2014. An expanded combined evidence approach to the *Gavialis* problem using geometric morphometric data from crocodylian braincases and eustachian systems. PLOS ONE 9, e105793.

Jiang, S.-X., Wang, X.-L., Meng, X. & Cheng, X. 2014. A new boreopterid pterosaur from the Lower Cretaceous of western Liaoning, China, with a reassessment of the phylogenetic relationships of the Boreopteridae. Journal of Paleontology 88, 823-828.

Kellner, A. W. A., Pinheiro, A. E. P. & Campos, D. A. 2014. A new sebecid from the Paleogene of Brazil and the crocodyliform radiation after the K-Pg boundary. PLOS ONE 9, e81386.

Manzig, P. C., Kellner, A. W. A., Weinschutz, L. C., Fragoso, C. E., Vega, C. S., Guimaraes, G. B., Godoy, L. C., Liccardo, A., Ricetti, J. H. Z. & Moura, C. C. 2014. Discovery of a rare pterosaur bone bed in a Cretaceous desert with insights on ontogeny and behavior of flying reptiles. PLOS ONE 9, e100005.

Martin, J. E., Lauprasert, K., Buffetaut, E., Liard, R. & Suteethorn, V. 2014. A large pholidosaurid in the Phu Kradung Formation of north-eastern Thailand. Palaeontology 57, 757-769.

Martin, J. E., Smith, T., De Broin, F. L., Escuillie, F. & Delfino, M. 2014. Late Palaeocene eusuchian remains from Mont de Berru, France, and the origin of the alligatoroid Diplocynodon. Zoological Journal of the Linnean Society 172, 867-891.

Mukherjee, D. & Ray, S. 2014. A new *Hyperodapedon* (Archosauromorpha, Rhynchosauria) from the Upper Triassic of India: implications for rhynchosaur phylogeny. Palaeontology 57, 1241-1276.

Nesbitt, S. J., Sidor, C. A., Angielczyk, K. D., Smith, R. M. H. & Tsuji, L. A. 2014. A new archosaur from the Manda beds (Anisian, Middle Triassic) of southern Tanzania and its implications for character state optimizations at Archosauria and Pseudosuchia. Journal of Vertebrate Paleontology 34, 1357-1382.

Pol, D., Nascimento, P. M., Carvalho, A. B., Riccomini, C., Pires-Domingues, R. A. & Zaher, H. 2014. A new notosuchian from the Late Cretaceous of Brazil and the phylogeny of advanced notosuchians. PLOS ONE 9, e93105.

Roberto-da-Silva, L., Desojo, J. B., Cabreira, S. F., Aires, A. S. S., Muller, R. T., Pacheco, C. P. & Dias-da-Silva, S. 2014. A new aetosaur from the Upper Triassic of the Santa Maria Formation, southern Brazil. Zootaxa 3764, 240-278.

Schoch, R. R. & Sues, H.-D. 2014. A new archosauriform reptile from the Middle Triassic (Ladinian) of Germany. Journal of Systematic Palaeontology 12, 113-131.

Sertich, J. J. W. and O'Connor, P. M. 2014. A new crocodyliform from the middle Cretaceous Galula Formation, southwestern Tanzania. Journal of Vertebrate Paleontology 34, 576-596.

Skutschas, P. P., Danilov, I. G., Kodrul, T. M. & Jin, J.-H. 2014. The first discovery of an alligatorid (Crocodylia, Alligatoroidea, Alligatoridae) in the Eocene of China. Journal of Vertebrate Paleontology 34, 471-476.

Sookias, R. B., Sennikov, A. G., Gower, D. J. & Butler, R. J. 2014. The monophyly of Euparkeriidae (Reptilia: Archosauriformes) and the origins of Archosauria: a revision of Dorosuchus neoetus from the mid-Triassic of Russia. Palaeontology 57, 1177-1202.

Sookias, R. B., Sullivan, C., Liu, J. & Butler, R. J. 2014. Systematics of putative euparkeriids (Diapsida: Archosauriformes) from the Triassic of China. PeerJ 2, e658.

Trotteyn, M. J. & Ezcurra, M. D. 2014. Osteology of *Pseudochampsa ischigualastensis gen. et comb. nov.* (Archosauriformes: Proterochampsidae) from the early Late Triassic Ischigualasto Formation of northwestern Argentina. PLOS One 9, e111388.

Vidovic, S. U. & Martill, D. M. 2014. *Pterodactylus scolopaciceps* Meyer, 1860 (Pterosauria, Pterodactyloidea) from the Upper Jurassic of Bavaria, Germany: the problem of cryptic pterosaur taxa in early ontogeny. PLOS One 9, e110646.

Vila Nova, B. C., Sayao, J. M., Neumann, V. H. M. L. & Kellner, A. W. A. 2014. Redescription of *Cearadactylus atrox* (Pterosauria, Pterodactyloidea) from the Early Cretaceous Romualdo Formation (Santana Group) of the Araripe Basin, Brazil. Journal of Vertebrate Paleontology 34, 126-134.

von Baczko, M. B., Desojo, J. B. & Pol, D. 2014. Anatomy and phylogenetic position of *Venaticosuchus rusconii* Bonaparte, 1970 (Archosauria, Pseudosuchia), from the Ischigualasto Formation (Late Triassic), La Rioja, Argentina. Journal of Vertebrate Paleontology 34, 1342-1356.

Wang, X.-L., Kellner, A. W. A., Jiang, S.-X., Wang, Q., Ma, Y.-X., Paidoula, Y., Cheng, X., Rodirgues, T., Meng, X., Zhang, J.-L., Li, N. & Zhou, Z.-H. 2014. Sexually dimorphic tridimensionally preserved pterosaurs and their eggs from China. Current Biology 24, 1323-1330.

Wang, X.-L., Rodrigues, T., Jiang, S.-X., Cheng, X. & Kellner, A. W. A. 2014. An Early Cretaceous pterosaur with an unusual mandibular crest from China and a potential novel feeding strategy. Scientific Reports 4, 6329.

Young, M. T. 2014. Filling the 'Corallian Gap': re-description of a metriorhynchid crocodylomorph from the Oxfordian (Late Jurassic) of Headington, England. Historical Biology 26, 80-90.

**2013**

Adams, T. L. 2013. A new neosuchian crocodyliform from the Lower Cretaceous (late Aptian) Twin Mountains Formation of North-Central Texas. Journal of Vertebrate Paleontology 33, 85-101.

Andres, B. & Myers, T. S. 2013. Lone Star pterosaurs. Earth and Environmental Science Transactions of the Royal Society of Edinburgh 103, 383-398.

Bennett, S. C. 2013. The phylogenetic position of the Pterosauria within the Archosauromorpha re-examined. Historical Biology 25, 545-563.

Bona, P., Riff, D. & Gasparini, Z. 2013. Late Miocene crocodylians from northeast Argentina: new approaches about the austral components of the Neogene South American crocodylian fauna. Earth and Environmental Science Transactions of the Royal Society of Edinburgh 103, 551-570.

Brochu, C. A. 2013. Phylogenetic relationships of Palaeogene ziphodont eusuchians and the status of *Pristichampsus* Gervais, 1853. Earth and Environmental Science Transactions of the Royal Society of Edinburgh 103, 521-550.

Conrad, J. L., Jenkins, K., Lehmann, T., Manthi, F. K., Peppe, D. J., Nightingale, S., Cossette, A., Dunsworth, H. M., Harcourt-Smith, W. E. H. & Mcnulty, K. P. 2013. New

specimens of *'Crocodylus' pigotti* (Crocodylidae) from Rusinga Island, Kenya, and generic reallocation of the species. Journal of Vertebrate Paleontology 33, 629-646.

Gao, K.-Q., Zhou, C.-F., Hou, L.-H. & Fox, R. C. 2013. Osteology and ontogeny of Early Cretaceous *Philydrosaurus* (Diapsida: Choristodera) based on new specimens from Liaoning Province, China. Cretaceous Research 45, 91-102.

Hastings, A. K., Bloch, J. I., Jaramillo, C. A., Rincon, A. F. & MacFadden, B. J. 2013. Systematics and biogeography of crocodylians from the Miocene of Panama. Journal of Vertebrate Paleontology 33, 239-263.

Herrera, Y., Gasparini, Z. & Fernandez, M. S. 2013. A new Patagonian species of *Cricosaurus* (Crocodyliformes, Thalattosuchia): first evidence of *Cricosaurus* in middle-upper Tithonian lithographic limestone from Gondwana. Palaeontology 56, 663-678.

Hungerbuhler, A., Mueller, B., Chatterjee, S. and Cunningham, D. P. 2013. Cranial anatomy of the Late Triassic phytosaur *Machaeroprosopus*, with the description of a new species from West Texas. Earth and Environmental Science Transactions of the Royal Society of Edinburgh 103, 269-312.

Iori, F. V., Marinho, T. D. S., Carvalho, I. D. S. & Campos, A. C. D. A. 2013. Taxonomic reappraisal of the sphagesaurid crocodyliform *Sphagesaurus montealtensis* from the Late Cretaceous Adamantina Formation of Sao Paulo State, Brazil. Zootaxa 3686, 183-200.

Irmis, R. B., Hutchison, J. H., Sertich, J. J. W. & Titus, A. L. 2013. Crocodyliforms from the Late Cretaceous of Grand Staircase-Escalante National Monument and vicinity, southern Utah, U.S.A. In Titus, A. L. and Loewen, M. A. (eds.) At the Top of the Grand Staircase: The Late Cretaceous of Southern Utah. Indiana University Press, Bloomington, p424-444.

Martin, J. E. & Vincent, P. 2013. New remains of *Machimosaurus hugii* von Meyer, 1837 (Crocodilia, Thalattosuchia) from the Kimmeridgian of Germany. Fossil Record 16, 179-196.

Mastrantonio, B. M., Schultz, C. L., Desojo, J. B. & Garcia, J. B. 2013. The braincase of *Prestosuchus chiniquensis* (Archosauria: Suchia). Geological Society of London Special Publications 379, 425-440.

Matsumoto, R., Buffetaut, E., Escuillie, F., Hervet, S. & Evans, S. E. 2013. New material of the choristodere *Lazarussuchus* (Diapsida, Choristodera) from the Paleocene of France. Journal of Vertebrate Paleontology 33, 319-339.

Montefeltro, F. C., Larsson, H. C. E., de Franca, M. A. G. & Langer, M. C. 2013. A new neosuchian with Asian affinities from the Jurassic of northeastern Brazil. Naturwissenschaften 100, 835-841.

Montefeltro, F. C., Bittencourt, J. S., Langer, M. C. & Schultz, C. L. 2013. Postcranial anatomy of the hyperodapedontine rhynchosaur *Teyumbaita sulcognathus* (Azevedo and Schultz, 1987) from the Late Triassic of Southern Brazil. Journal of Vertebrate Paleontology 33, 67-84.

Naish, D., Simpson, M. & Dyke, G. 2013. A new small-bodied azhdarchoid pterosaur from the Lower Cretaceous of England and its implications for pterosaur anatomy, diversity and phylogeny. PLOS One 8, e58451.

Nesbitt, S. J., Butler, R. J. & Gower, D. J. 2013. A new archosauriform (Reptilia: Diapsida) from the Manda Beds (Middle Triassic) of Southwestern Tanzania. PLOS ONE 8, e72753.

Parrilla-Bel, J., Young, M. T., Moreno-Azanza, M. & Canudo, J. I. 2013. The first metriorhynchid crocodylomorph from the Middle Jurassic of Spain, with implications for evolution of the subclade Rhacheosaurini. PLOS One 8, e54275.

Peecook, B. R., Sidor, C. A., Nesbitt, S. J., Smith, R. M. H., Steyer, J. S. & Angielczyk, K. D. 2013. A new silesaurid from the upper Ntawere Formation of Zambia (Middle Triassic) demonstrates the rapid diversification of Silesauridae (Avemetatarsalia, Dinosauriformes). Journal of Vertebrate Paleontology 33, 1127-1137.

Pol, D., Rauhut, O. W. M., Lecuona, A., Leardi, J. M., Xu, X. & Clark, J. M. 2013. A new fossil from the Jurassic of Patagonia reveals the early basicranial evolution and the origins of Crocodyliformes. Biological Reviews 88, 862-872.

Pritchard, A. C., Turner, A. H., Allen, E. R. & Norell, M. A. 2013. Osteology of a North American goniopholidid (*Eutretauranosuchus delfsi*) and palate evolution in Neosuchia. American Museum Novitates 3783, 1-56.

Rodrigues, T. & Kellner, A. W. A. 2013. Taxonomic review of the Ornithocheirus complex (Pterosauria) from the Cretaceous of England. ZooKeys 308, 1-112.

Scheyer, T. M., Aguilera, O. A., Delfino, M., Fortier, D. C., Carlini, A. A., Sanchez, R., Carrillo-Briceno, J. D., Quiroz, L. & Sanchez-Villagra, M. R. 2013. Crocodylian diversity peak and extinction in the late Cenozoic of the northern Neotropics. Nature Communications 4, 1907.

Sues, H.-D. & Schoch, R. R. 2013. Reassessment of *cf. Halticosaurus orbitoangulatus* from the Upper Triassic (Norian) of Germany - a pseudosuchian, not a dinosaur. Zoological Journal of the Linnean Society 168, 859-872.

Toljagic, O. & Butler, R. J. 2013. Triassic-Jurassic mass extinction as trigger for the Mesozoic radiation of crocodylomorphs. Biology Letters 9, 20130095.

Wang, R.-F., Xu, S.-C., Wu, X.-C., Li, C. & Wang, S.-Z. 2013. A new specimen of *Shansisuchus shansisuchus* Young, 1964 (Diapsida: Archosauriformes) from the Triassic of Shanxi, China. Act Geologica Sinica 87, 1185-1197.

Young, M. T., de Andrade, M. B., Brusatte, S. L., Sakamoto, M. & Liston, J. 2013. The oldest known metriorhynchid super-predator: a new genus and species from the Middle Jurassic of England, with implications for serration and mandibular evolution in predacious clades. Journal of Systematic Palaeontology 11, 475-513.

Young, M. T., Andrade, M. B., Etches, S. & Beatty, B. L. 2013. A new metriorhynchid crocodylomorph from the Lower Kimmeridge Clay Formation (Late Jurassic) of England, with implications for the evolution of dermatocranium ornamentation in Geosaurini. Zoological Journal of the Linnean Society 169, 820-848.

**2012**

Brochu, C. A. & Storrs, G. W. 2012. A giant crocodile from the Plio-Pleistocene of Kenya, the phylogenetic relationships of Neogene African crocodylines, and the antiquity of Crocodylus in Africa. Journal of Vertebrate Paleontology 32, 587-602.

Brochu, C. A., Parris, D. C, Grandstaff, B. S., Denton, R. K. & Gallagher W. B. 2012. A new species of *Borealosuchus* (Crocodyliformes, Eusuchia) from the Late Cretaceous-early Paleogene of New Jersey. Journal of Vertebrate Paleontology 32, 105-116.

Bronzati, M., Montefeltro, F. C. & Langer, M. C. 2012. A species-level supertree of Crocodyliformes. Historical Biology 24, 598-606.

Delfino, M. & Smith, T. 2012. Reappraisal of the morphology and phylogenetic relationships of the middle Eocene alligatoroid *Diplocynodon deponiae* (Frey, Laemmert, and Riess, 1987) based on a three-dimensional specimen. Journal of Vertebrate Paleontology 32, 1358-1369.

Desojo, J. B., Ezcurra, M. D. & Kischlat, E. E. 2012. A new aetosaur genus (Archosauria: Pseudosuchia) from the early Late Triassic of southern Brazil. Zootaxa 3166, 1-33.

Dilkes, D. and Arcucci, A. 2012. *Proterochampsa barrionuevoi* (Archosauriformes: Proterochampsia) from the Late Triassic (Carnian) of Argentina and a phylogenetic analysis of *Proterochampsia*. Palaeontology 55, 853-885.

Holliday, C. M. & Gardner, N. M. 2012. A new eusuchian crocodyliform with novel cranial integument and its significance for the origin and evolution of Crocodylia. PLoS ONE 7, e30471.

Leardi, J. M., Pol, D. & Fernandez, M. S. 2012. The antorbital fenestra of Metriorhynchidae (Crocodyliformes, Thalattosuchia): testing its homology within a phylogenetic framework. Journal of Vertebrate Paleontology 32, 490-494.

Li, C., Wu, X.-C., Zhao, L.-J., Sato, T. & Wang, L.-T. 2012. A new archosaur (Diapsida, Archosauriformes) from the marine Triassic of China. Journal of Vertebrate Paleontology 32, 1064-1081.

Lu, J.-C., Pu, H.-Y., Xu, L., Wu, Y. & Wei, X.-F, 2012. Largest toothed pterosaur skull from the Early Cretaceous Yixian Formation of western Liaoning, China, with comments on the family Boreopteridae. Acta Geologica Sinica 86, 287-293.

Lu, J.-C., Unwin, D. M., Zhao, B., Gao, C.-L. & Shen, C.-Z. 2012. A new rhamphorhynchid (Pterosauria: Rhamphorhynchidae) from the Middle/Upper Jurassic of Qinglong, Hebei Province, China. Zootaxa 3158, 1-19.

Martin, J. E. & Buffetaut, E. 2012. The maxillary depression of Pholidosauridae: an anatomical study. Journal of Vertebrate Paleontology 32, 1442-1446.

Pereda-Suberbiola, X., Knoll, F., Ruiz-Omenaca, J. I., Company, J. & Torcida Fernandez-Baldor, F. 2012. Reassessment of *Prejanopterus curvirostris*, a basal pterodactyloid pterosaur from the Early Cretaceous of Spain. Acta Geologica Sinica 86, 1389-1401.

Pol, D., Leardi, J. M., Lecuona, A. & Krause, M. 2012. Postcranial anatomy of Sebecus icaeorhinus (Crocodyliformes, Sebecidae) from the Eocene of Patagonia. Journal of Vertebrate Paleontology 32, 328-354.

St John, J. A., Braun, E. L., Isberg, S. R., Miles, L. G., Chong, A. Y., Gongora, J., Dalzell, P., Moran, C., Bed'Hom, B., Abzhanov, A., Burgess, S. C., Cooksey, A. M., Castoe, T. A., Crawford, N. G., Densmore, L. D., Drew, J. C., Edwards, S. V., Faircloth, B. C., Fujita, M. K., Greenwold, M. J., Hoffmann, F. G., Howard, J. M., Iguchi, T., Janes, D. E., Khan, S. Y., Kohno, S., de Koning, A. P. J., Lance, S. L., McCarthy, F. M., McCormack, J. E., Merchant, M. E., Peterson, D. G., Pollock, D. D., Pourmand, N., Raney, B. J., Roessler, K. A., Sanford, J. R., Sawyer, R. H., Schmidt, C. J., Triplett, E. W., Tuberville, T. D., Venegas-Anaya, M., Howard, J. T., Jarvis, E. D., Guillette, L. J., Glenn, T. C., Green, R. E. & Ray, D. A. 2012. Sequencing three crocodilian genomes to illuminate the evolution of archosaurs and amniotes. Genome Biology 13, 415.

Stocker, M. R. 2012. A new phytosaur (Archosauriformes, Phytosauria) from the Lot's Wife beds (Sonsela Member) within the Chinle Formation (Upper Triassic) of Petrified Forest National Park, Arizona, Journal of Vertebrate Paleontology 32, 573-586.

Trotteyn, M. J. & Haro, J. A. 2012. The braincase of *Chanaresuchus ischigualastensis* (Archosauriformes) from the Late Triassic of Argentina. Journal of Vertebrate Paleontology 32, 867-882.

Vullo, R., Marugan-Lobon, J., Kellner, A. W. A., Buscalioni, A. D., Gomez, B., de la Fuente, M. & Moratalla, J. J. 2012. A new crested pterosaur from the Early Cretaceous of Spain: the first European tapejarid (Pterodactyloidea: Azhdarchoidea). PLOS ONE 7, e38900.

Wang, X.-L., Kellner, A. W. A., Jiang, S.-X. & Cheng, X. 2012. New toothed flying reptile from Asia: close similarities between early Cretaceous pterosaur faunas from China and Brazil. Naturwissenschaften 99, 249-257.

Witton, M. P. 2012. New insights into the skull of Istiodactylus latidens (Ornithocheiroidea, Pterodactyloidea). PLoS ONE 7, e33170.

Young, M. T., Brusatte, S. L., Andrade, M. B., Desojo, J. B., Beatty, B. L., Steel, L., Fernandez, M. S., Sakamoto, M., Ruiz-Omenaca, J. I. & Schoch, R. R. 2012. The cranial osteology and feeding ecology of the metriorhynchid crocodylomorph genera *Dakosaurus* and *Plesiosuchus* from the Late Jurassic of Europe. PLoS One 7, e44895.

**2011**

Andrade, M. D., Edmonds, R., Benton, M. J. & Schouten R. 2011. A new Berriasian species of *Goniopholis* (Mesoeucrocodylia, Neosuchia) from England, and a review of the genus. Zoological Journal of the Linnean Society 163, S66-S108.

Brochu, C. A. 2011. Phylogenetic relationships of *Necrosuchus ionensis* Simpson, 1937 and the early history of caimanines. Zoological Journal of the Linnean Society 163, S228-S256.

Buscalioni, A. D., Piras, P., Vullo, R., Signore, M. & Barbera, C. 2011. Early Eusuchia Crocodylomorpha from the vertebrate-rich Plattenkalk of Pietraroia (Lower Albian, southern Apennines, Italy). Zoological Journal of the Linnean Society 163, S199-S227.

Butler, R. J., Brusatte, S. L., Andres, A. & Benson, R. B. J. 2011. How do geological sampling biases affect studies of morphological evolution in deep time? A case study of pterosaur (Reptilia: Archosauria) disparity. Evolution 66, 147-162.

Cau, A. & Fanti, F. 2011. The oldest known metriorhynchid crocodylian from the Middle Jurassic of North-eastern Italy: *Neptunidraco ammoniticus gen. et sp. nov.* Gondwana Research 19, 550-565.

Clark, J. M. 2011. A new shartegosuchid crocodyliform from the Upper Jurassic Morrison Formation of western Colorado. Zoological Journal of the Linnean Society 163, S152-S172.

Desojo, J. B., Ezcurra, M. D. & Schultz, C. L. 2011. An unusual new archosauriform from the Middle-Late Triassic of southern Brazil and the monophyly of Doswelliidae. Zoological Journal of the Linnean Society 161, 839-871.

Fortier, D., Perea, D. & Schultz, C. 2011. Redescription and phylogenetic relationships of *Meridiosaurus vallisparadisi*, a pholidosaurid from the Late Jurassic of Uruguay. Zoological Journal of the Linnean Society 163, S257-S272.

Franca, M. A., Ferigolo, J. & Langer, M. C. 2011. Associated skeletons of a new Middle triassic "Rauisuchia" from Brazil. Naturwissenschaften 98, 389-395.

Hastings, A. K., Bloch, J. I. & Jaramillo, C. A. 2011. A new longirostrine dyrosaurid (Crocodylomorpha, Mesoeucrocodylia) from the Paleocene of north-eastern Colombia: biogeographic and behavioural implications for New-World Dyrosauridae. Palaeontology 54, 1095-1116.

Iori, F. V. & Carvalho, I. S. 2011. *Caipirasuchus paulistanus*, a new sphagesaurid (Crocodylomorpha, Mesoeucrocodylia) from the Adamantina Formation (Upper Cretaceous, Turonian-Santonian), Bauru Basin, Brazil. Journal of Vertebrate Paleontology 31, 1255-1264.

Martin, J. E. & Gross, M. 2011. Taxonomic ciarification of *Diplocynodon* Pomel, 1847 (Crocodilia) from the Miocene of Styria, Austria. Neues Jahrbuch fur Geologie und Palaontologie Abhandlungen 261, 177-193.

Meredith, R. W., Hekkala, E. R., Amato, G. & Gatesy, J. 2011. A phylogenetic hypothesis for *Crocodylus* (Crocodylia) based on mitochondrial DNA: evidence for a trans-Atlantic voyage from Africa to the New World. Molecular Phylogenetics and Evolution 60, 183-191.

Montefeltro, F. C., Larsson, H. C. E. & Langer, M. C. 2011. A new baurusuchid (Crocodyliformes, Mesoeucrocodylia) from the Late Cretaceous of Brazil and the phylogeny of Baurusuchidae. PLoS ONE 6, e21916.

Nascimento, P. M. & Zaher, H. 2011. The skull of the Upper Cretaceous baurusuchid crocodile *Baurusuchus albertoi* Nascimento & Zaher 2010, and its phylogenetic affinities. Zoological Journal of the Linnean Society 163, S116-S131.

Pinheiro, F. L., Fortier, D. C., Schultz, C. L., Andrade, J. A. F. G. & Bantim, R. A. M. 2011. New information on the pterosaur *Tupandactylus imperator*, with comments on the relationships of Tapejaridae. Acta Palaeontologica Polonica 56, 567-580.

Pol, D. & Powell, J. E. 2011. A new sebecid mesoeucrocodylian from the Rio Loro Formation (Palaeocene) of north-western Argentina. Zoological Journal of the Linnean Society 163, S7-S36.

Puertolas, E., Canudo, J. I. & Cruzado-Caballero, P. 2011. A new crocodylian from the late Maastrichtian of Spain: implications for the initial radiation of crocodyloids. PLoS ONE 6, e20011.

Riff, D. & Kellner, A. W. A. 2011. Baurusuchid crocodyliforms as theropod mimics: clues from the skull and appendicular morphology of *Stratiotosuchus maxhechti* (Upper Cretaceous of Brazil). Zoological Journal of the Linnean Society 163, S37-S56.

Soto, M., Pol, D. & Perea, D. 2011. A new specimen of *Uruguaysuchus aznarezi* (Crocodyliformes: Notosuchia) from the middle Cretaceous of Uruguay and its phylogenetic relationships. Zoological Journal of the Linnean Society 163, S173-S198.

Young, M. T., Bell, M. A., De Andrade, M. B. & Brusatte, S. L. 2011. Body size estimation and evolution in metriorhynchid crocodylomorphs: implications for species diversification and niche partitioning. Zoological Journal of the Linnean Society 163, 1199-1216.

Zhang, M., Wang, Y.-H., Yan, P. & Wu, X.-B. 2011. Crocodilian phylogeny inferred from twelve mitochondrial protein-coding genes, with new complete mitochondrial genomic sequences for *Crocodylus acutus* and *Crocodylus novaeguineae*. Molecular Phylogenetics and Evolution 60, 62-67.

**2010**

Andrade, M. B., Young, M. T., Desojo, J. B. & Brusatte, S. L. 2010. The evolution of extreme hypercarnivory in Metriorhynchidae (Mesoeucrocodylia: Thalattosuchia) based on evidence from microscopic denticle morphology. Journal of Vertebrate Paleontology 30, 1451-1465.

Andres, B., Clark, J. M. & Xu, X. 2010. A new rhamphorhynchid pterosaur from the Upper Jurassic of Xinjiang, China, and the phylogenetic relationships of basal pterosaurs. Journal of Vertebrate Paleontology 30, 163-187.

Brochu, C. A. 2010. A new alligatorid from the Lower Eocene Green River Formation of Wyoming and the origin of caimans. Journal of Vertebrate Paleontology 30, 1109-1126.

Brochu, C. A., Njau, J., Blumenschine, R. J. and Densmore, L. D. 2010. A new horned crocodile from the Plio-Pleistocene hominid sites at Olduvai Gorge, Tanzania. PLoS ONE 5, e9333.

Delfino, M. and De Vos, J. 2010. A revision of the *Dubois crocodylians*, *Gavialis bengawanicus* and *Crocodylus ossifragus*, from the Pleistocene Homo erectus Beds of Java. Journal of Vertebrate Paleontology 30, 427-441.

Ezcurra, M. D., Lecuona, A. and Martinelli, A. 2010. A new basal archosauriform diapsid from the Lower Triassic of Argentina. Journal of Vertebrate Paleontology 30, 1433-1450.

Feng, G., Wu, X.-B., Yan, P. and Li, X.-Q. 2010. Two complete mitochondrial genomes of *Crocodylus* and implications for crocodilians phylogeny. Amphibia-Reptilia 31, 299-309.

Hastings, A. K., Bloch, J. I., Cadena, E. A. & Jaramillo, C. A. 2010. A new small short-snouted dyrosaurid (Crocodylomorpha, Mesoeucrocodylia) from the Paleocene of northeastern Colombia. Journal of Vertebrate Paleontology 30, 139-162.

Lu, J.-C., Unwin, D. M., Jin, X.-S., Liu, Y.-Q. & Ji, Q. 2010. Evidence for modular evolution in a long-tailed pterosaur with a pterodactyloid skull. Proceedings of the Royal Society of London B 277, 383-389.

Martin, J. E. 2010. A new species of *Diplocynodon* (Crocodylia, Alligatoroidea) from the Late Eocene of the Massif Central, France, and the evolution of the genus in the climatic context of the Late Palaeogene. Geological Magazine 147, 596-610.

Martin, J. E. & Lauprasert, K. 2010. A new primitive alligatorine from the Eocene of Thailand: relevance of Asiatic members to the radiation of the group. Zoological Journal of the Linnean Society, 158, 608-628.

Martin, J. E., Rabi, M. & Csiki, Z. 2010. Survival of *Theriosuchus* (Mesoeucrocodylia: Atoposauridae) in a Late Cretaceous archipelago: a new species from the Maastrichtian of Romania. Naturwissenschaften 97, 845-854.

Meganathan, P. R., Dubey, B., Batzer, M. A., Ray, D. A. & Haque, I. 2010. Molecular phylogenetic analyses of genus *Crocodylus* (Eusuchia, Crocodylia, Crocodylidae) and the taxonomic position of *Crocodylus porosus*. Molecular Phylogenetics and Evolution 57, 393-402.

O'Connor, P. M., Sertich, J. J. W., Stevens, N. J., Roberts, E. M., Gottfried, M. D., Hieronymus, T. L., Jinnah, Z. A., Ridgely, R., Ngasala, S. E. & Temba, J. 2010. The evolution of mammal-like crocodyliforms in the Cretaceous Period of Gondwana. Nature 466, 748-751.

Renesto, S., Spielmann, J. A., Lucas, S. G. & Spagnoli, G. T. 2010. The taxonomy and paleobiology of the Late Triassic (Carnian-Norian: Adamanian-Apachean) drepanosaurs (Diapsida: Archosauromorpha: Drepanosauromorpha). Bulletin of the New Mexico Museum of Natural History & Science 46, 1-81.

Smith, D. K., Allen, E. R., Sanders, R. K. & Stadtman, K. L. 2010. A new specimen of *Eutretauranosuchus* (Crocodyliformes; Goniopholididae) from Dry Mesa, Colorado. Journal of Vertebrate Paleontology 30, 1466-1477.

Stocker, M. R., 2010. A new taxon of phytosaur (Archosauria: Pseudosuchia) from the Late Triassic (Norian) Sonsela Member (Chinle Formation) in Arizona, and a critical reevaluation of *Leptosuchus* Case, 1922. Palaeontology 53, 997-1022.

Turner, A. H. & Sertich, J. J. W. 2010. Phylogenetic history of *Simosuchus clarki* (Crocodyliformes: Notosuchia) from the Late Cretaceous of Madagascar. Journal of Vertebrate Paleontology 30, s177-s236.

**2009**

Bhullar, B.-A. S. & Bever, G. S. 2009. An archosaur-like laterosphenoid in early turtles (Reptilia: Pantestudines). Breviora 518, 1-11.

Borsuk-Bialynicka, M. & Evans, S. E. 2009. A long-necked archosauromorph from the Early Triassic of Poland. Palaeontologia Polonica 65, 203-234.

Dalla Vecchia, F. M. 2009. Anatomy and systematics of the pterosaur *Carniadactylus gen. n. rosenfeldi* (Dalla Vecchia, 1995). Rivista Italiana di Paleontologia e Stratigrafia 115, 159-188.

Dalla Vecchia, F. M. 2009. The first Italian specimen of *Austriadactylus cristatus* (Diapsida, Pterosauria) from the Norian (Upper Triassic) of the Carnic Prealps. Rivista Italiana di Paleontologia e Stratigrafia 115, 291-304.

Delfino, M. & Smith, T. 2009. A reassessment of the morphology and taxonomic status of *'Crocodylus depressifrons'* Blainville, 1855 (Crocodylia, Crocodyloidea) based on the Early Eocene remains from Belgium. Zoological Journal of the Linnean Society 156, 140-167.

Dilkes, D. & Sues, H.-D. 2009. Redescription and phylogenetic relationships of *Doswellia kaltenbachi* (Diapsida: Archosauriformes) from the Upper Triassic of Virginia. Journal of Vertebrate Paleontology 29, 58-79.

Eaton, M. J., Martin, A., Thorbjarnarson, J. & Amato, G. 2009. Species-level diversification of African dwarf crocodiles (genus *Osteolamus*): a geographic and phylogenetic perspective. Molecular Phylogenetics and Evolution 50, 496-506.

Fortier, D. C. & Schultz, C. L. 2009. A new neosuchian crocodylomorph (Crocodyliformes, Mesoeucrocodylia) from the Early Cretaceous of North-East Brazil. Palaeontology 52, 991-1007.

Gottmann-Quesada, A. & Sander, P. M. 2009. A redescription of the early archosauromorph *Protorosaurus speneri* Meyer, 1832, and its phylogenetic relationships. Palaeontographica Abteilung A 287, 123-220.

Jouve, S. 2009. The skull of *Teleosaurus cadomensis* (Crocodylomorpha; Thalattosuchia), and phylogenetic analysis of Thalattosuchia. Journal of Vertebrate Paleontology 29, 88-102.

Lauprasert, K., Cuny, G., Thirakhupt, K. & Suteethorn, V. 2009. *Khoratosuchus jintasakuli gen. et sp. nov*., an advanced neosuchian crocodyliform from the Early Cretaceous (Aptian-Albian) of NE Thailand. Geological Society, London, Special Publications 315, 175-187.

Leardi, J. M. & Pol, D. 2009. The first crocodyliform from the Chubut Group (Chubut Province, Argentina) and its phylogenetic position within basal Mesoeucrocodylia. Cretaceous Research 30, 1376-1386.

Matsumoto, R., Suzuki, S., Tsogtbaatar, K. and Evans, S. E. 2009. New material of the enigmatic reptile *Khurendukhosaurus* (Diapsida: Choristodera) from Mongolia. Naturwissenschaften 96, 233-242.

Nesbitt, S. J., Stocker, M. R., Small, B. J. & Downs, A. 2009. The osteology and relationships of *Vancleavea campi* (Reptilia: Archosauriformes). Zoological Journal of the Linnean Society 157, 814-864.

Novas, F. E., Pais, D. F., Pol, D., Carvalho, I. S., Scanferla, A., Mones, A. & Riglos, M. S. 2009. Bizarre notosuchian crocodyliform with associated eggs from the Upper Cretaceous of Bolivia. Journal of Vertebrate Paleontology 29, 1316-1320.

Pol, D. & Gasparini, Z. 2009. Skull anatomy of *Dakosaurus andiniensis* (Thalattosuchia: Crocodylomorpha) and the phylogenetic position of Thalattosuchia. Journal of Systematic Palaeontology 7, 163-197.

Pol, D., Turner, A. H. & Norell, M. A. 2009. Morphology of the Late Cretaceous crocodylomorph *Shamosuchus djadochtaensis* and a discussion of neosuchian phylogeny as related to the origin of Eusuchia. Bulletin of the American Museum of Natural History 324, 1-103.

Sereno, P. C. & Larsson, H. C. E. 2009. Cretaceous Crocodyliforms from the Sahara. ZooKeys 28, 1-143.

Shan, H.-Y., Wu, X.-C., Cheng, Y.-N. & Sato, T. 2009. A new tomistomine (Crocodylia) from the Miocene of Taiwan. Canadian Journal of Earth Sciences 46, 529-555.

Wang, X.-L., Kellner, A. W. A., Jiang, S.-X. & Meng, X. 2009. An unusual long-tailed pterosaur with elongated neck from western Liaoning of China. Anais da Academia Brasileira de Ciências 81, 793-812.

Willis, R. E. 2009. Transthyretin gene (TTR) intron 1 elucidates crocodylian phylogenetic relationships. Molecular Phylogenetics and Evolution 53, 1049-1054.

Young, M. T. & Andrade, M. D. 2009. What is *Geosaurus*? Redescription of Geosaurus giganteus (Thalattosuchia: Metriorhynchidae) from the Upper Jurassic of Bayern, Germany. Zoological Journal of the Linnean Society 157, 551-585.

**2008**

Andrade, M. B. & Bertini, R. J. 2008. Morphological and anatomical observations about M*ariliasuchus amarali* and *Notosuchus terrestris* (Mesoeucrocodylia) and their relationships with other South American notosuchians. Arquivos do Museu Nacional Rio de Janeiro 66, 5-62.

Andrade, M. B. & Bertini, R. J. 2008. A new Sphagesaurus (Mesoeucrocodylia: Notosuchia) from the Upper Cretaceous of Monte Alto City (Bauru Group, Brazil), and a revision of the Sphagesauridae. Historical Biology 20, 101-136.

Andres, B. & Ji, Q. 2008. A new pterosaur from the Liaoning Province of China, the phylogeny of the Pterodactyloidea, and convergence in their cervical vertebrae. Palaeontology 51, 453-469.

Barbosa, J. A., Kellner, A. W. A. & Viana, M. S. S. 2008. New dyrosaurid crocodylomorph and evidences for faunal turnover at the K-P transition in Brazil. Proceedings of the Royal Society of London B 275, 1385-1391.

Brusatte, S. L., Benton, M. J., Ruta, M. & Lloyd, G. T. 2008. Superiority, competition, and opportunism in the evolutionary radiation of dinosaurs. Science 321, 1485-1488.

Delfino, M. & Rook, L. 2008. African crocodylians in the late Neogene of Europe: a revision of *Crocodylus bambolii* Ristori, 1890.Journal of Paleontology 82, 336-343.

Delfino, M., Martin, J. E. & Buffetaut, E. 2008. A new species of *Acynodon* (Crocodylia) from the Upper Cretaceous (Santonian-Campanian) of Villaggio del Pescatore, Italy. Palaeontology 51, 1091-1106.

Delfino, M., Codrea, V., Folie, A., Dica, P., Godefroit, P. & Smith, T. 2008. A complete skull of *Allodaposuchus precedens* Nopcsa, 1928 (Eusuchia) and a reassessment of the morphology of the taxon based on the Romanian remains. Journal of Vertebrate Paleontology 28, 111-122.

Fiorelli, L. & Calvo, J. O. 2008. New remains of *Notosuchus terrestris* Woodward, 1896 (Crocodyliformes: Mesoeucrocodylia) from Late Cretaceous of Neuquen, Patagonia, Argentina. Arquivos do Museu Nacional Rio de Janeiro 66, 83-124.

Hill, R. V., McCartney, J. A., Roberts, E. M., Bouare, M. L., Sissoko, F. & O'Leary, M. A. 2008. Dyrosaurid (Crocodyliformes, Mesoeucrocodylia) fossils from the Upper Cretaceous and Paleogene of Mali: implications for phylogeny and survivorship across the K-T boundary. American Museum Novitates 3631, 1-19.

Hone, D. W. E. & Benton, M. J. 2008. A new genus of rhynchosaur from the Middle Triassic of south-west England. Palaeontology 51, 95-115.

Jouve, S., Bouya, B. & Amaghzaz, M. 2008. A long-snouted dyrosaurid (Crocodyliformes, Mesoeucrocodylia) from the Paleocene of Morocco: phylogenetic and palaeobiogeographic implications. Palaeontology 51, 281-294.

Jouve, S., Bardet, N., Jalil, N.-E., Pereda Suberbiola, X., Bouya, B. & Amaghzaz, M. 2008. The oldest African crocodylian: phylogeny, paleobiogeography, and differential survivorship of marine reptiles through the Crteaceous-Tertiary boundary. Journal of Vertebrate Paleontology 28, 409-421.

Lu, J.-C., Unwin, D. M., Xu, L. & Zhang, X.-L. 2008. A new azhdarchoid pterosaur from the Lower Cretaceous of China and its implications for pterosaur phylogeny and evolution. Naturwissenschaften 95, 891-897.

Martin, J. E. & Buffetaut, E. 2008. *Crocodilus affuvelensis* Matheron, 1869 from the Late Cretaceous of southern France: a reassessment. Zoological Journal of the Linnean Society 152, 567-580.

Parker, W. G. & Barton, B. J. 2008. New information on the Upper Triassic archosauriform *Vancleavea campi* based on new material from the Chinle Formation of Arizona. Palaeontologia Electronica 11, 1-20.

Parker, W. G., Stocker, M. R. & Irmis, R. B. 2008. A new desmatosuchine aetosaur (Archosauria: Suchia) from the Upper Triassic Tecovas Formation (Dockum Group) of Texas. Journal of Vertebrate Paleontology 28, 692-701.

Skutschas, P. P. 2008. A choristoderan reptile from the Lower Cretaceous of Transbaikalia, Russia. Neues Jahrbuch fur Geologie und Palaontologie Abhandlungen 247, 63-78.

Spielmann, J. A., Lucas, S. G., Rineheart, L. F. & Heckert, A. B. 2008. The Late Triassic archosauromorph *Trilophosaurus*. Bulletin of the New Mexico Museum of Natural History & Science 43, 1-24.

Turner, A. H. & Buckley, G. A. 2008. *Mahajangasuchus insignis* (Crocodyliformes: Mesoeucrocodylia) cranial anatomy and new data on the origin of the eusuchian-style palate. Journal of Vertebrate Paleontology 28, 382-408.

Wang, X.-L., Kellner, A. W. A., Zhou, Z.-H. & Campos, D. A. 2008. Discovery of a rare arboreal forest-dwelling flying reptile (Pterosauria, Pterodactyloidea) from China. Proceedings of the National Academy of Sciences USA 105, 1983-1987.

Wilkinson, L. E., Young, M. T. & Benton, M. J. 2008. A new metriorhynchid crocodilian (Mesoeucrocodylia: Thalattosuchia) from the Kimmeridgian (Upper Jurassic) of Wiltshire, UK. Palaeontology 51, 1307-1333.

**2007**

Bennett, S. C. 2007. A second specimen of the pterosaur *Anurognathus ammoni.* Palaontologische Zeitschrift 81, 376-398.

Bona, P. 2007. Una nueva especie de *Eocaiman* Simpson (Crocodylia, Alligatoridae) del Paleoceno Inferior de Patagonia. Ameghiniana 44, 435-445.

Brochu, C. A. 2007. Morphology, relationships, and biogeographical significance of an extinct horned crocodile (Crocodylia, Crocodylidae) from the Quaternary of Madagascar. Zoological Journal of the Linnean Society 150, 835-863.

Brochu, C. A. 2007. Systematics and taxonomy of Eocene tomistomine crocodylians from Britain and northern Europe. Palaeontology 50, 917-928.

Fiorelli, L. E. & Calvo, J. O. 2007. The first "protosuchian" (Archosauria: Crocodyliformes) from the Cretaceous (Santonian) of Gondwana. Arquivos do Museu Nacional Rio de Janeiro 65, 417-459.

Hugall, A. F., Foster, R. & Lee, M. S. Y. 2007. Calibration choice, rate smoothing, and the pattern of tetrapod diversification according to the long nuclear gene RAG-1. Systematic Biology 56, 543-563.

Larsson, H. C. E. & Sues, H.-D. 2007. Cranial osteology and phylogenetic relationships of *Hamadasuchus rebouli* (Crocodyliformes: Mesoeucrocodylia) from the Cretaceous of Morocco. Zoological Journal of the Linnean Society 149, 533-567.

Lauprasert, K., Cuny, G., Buffetaut, E., Suteethorn, V. & Thirakhupt, K. 2007. *Siamosuchus phuphokensis*, a new goniopholidid from the Early Cretaceous (ante-Aptian) of northeastern Thailand. Bulletin de la Societe Geologique de France 178, 201-216.

Matsumoto, R., Evans, S. E. & Manabe, M. 2007. The choristoderan reptile *Monjurosuchus* from the Early Cretaceous of Japan. Acta Palaeontologica Polonica 52, 329-350.

Osi, A., Clark, J. M. & Weishampel, D. B. 2007. First report on a new basal eusuchian crocodyliform with multicusped teeth from the Upper Cretaceous (Santonian) of Hungary. Neues Jahrbuch fur Geologie und Palaontologie Abhandlungen 243, 169-177.

Parker, W. G. 2007. Reassessment of the aetosaur *'Desmatosuchus' chamaensis* with a reanalysis of the phylogeny of the Aetosauria (Archosauria: Pseudosuchia). Journal of Systematic Palaeontology 5, 41-68.

Piras, P., Delfino, M., Del favero, L. & Kotsakis, T. 2007. Phylogenetic position of the crocodylian *Megadontosuchus arduini* and tomistomine palaeobiogeography. Acta Palaeontologica Polonica 52, 315-328.

Roos, J., Aggarwal, R. K. & Janke, A. 2007. Extended mitogenomic phylogenetic analyses yield new insight into crocodylian evolution and their survival of the Cretaceous-Tertiary boundary. Molecular Phylogenetics and Evolution 45, 663-673.

Snyder, D. 2007. Morphology and systematics of two Miocene alligators from Florida, with a discussion of *Alligator* biogeography. Journal of Paleontology 81, 917-928.

Velez-Juarbe, J., Brochu, C. A. & Santos, H. 2007. A gharial from the Oligocene of Puerto Rico: transoceanic dispersal in the history of a non-marine reptile. Proceedings of the Royal Society of London B 274, 1245-1254.

Weinbaum, J. C. & Hungerbuhler, A. 2007. A revision of *Poposaurus gracili*s (Archosauria: Suchia) based on two new specimens from the Late Triassic of the southwestern U.S.A. Palaontologische Zeitschrift 81, 131-145.

**2006**

Aguilera, O. A., Riff, D. & Bocquentin-Villanueva, J. 2006. A new giant *Purussaurus* (Crocodyliformes, Alligatoridae) from the Upper Miocene Urumaco Formation, Venezuela. Journal of Systematic Palaeontology 4, 221-232.

Brochu, C. A. 2006. Osteology and phylogenetic significance of *Eosuchus minor* (Marsh, 1870) new combination, a longirostrine crocodylian from the Late Paleocene of North America. Journal of Paleontology 80, 162-186.

Brochu, C. A. 2006. A new miniature horned crocodile from the Quaternary of Aldabra Atoll, Western Indian Ocean. Copeia 2006, 149-158.

Gasparini, Z., Pol, D. & Spalletti, L. A. 2006. An unusual marine crocodyliform from the Jurassic-Cretaceous boundary of Patagonia. Science 311, 70-73.

Gower, D. J. & Nesbitt, S. J. 2006. The braincase of *Arizonasaurus babbitti* - further evidence for the non-monophyly of 'rauisuchian' archosaurs. Journal of Vertebrate Paleontology 26, 79-87.

Jouve, S., Iarochene, M., Bouya, B. & Amaghzaz, M. 2006. A new species of *Dyrosaurus* (Crocodylomorpha, Dyrosauridae) from the Early Eocene of Morocco: phylogenetic implications. Zoological Journal of the Linnean Society 148, 603-656.

Jouve, S., Iarochene, M., Bouya, B. & Amaghzaz, M. 2006. New material of *Argochampsa krebsi* (Crocodylia: Gavialoidea) from the Lower Paleocene of the Oulad Abdoun Basin (Morocco): phylogenetic implications. Geobios 39, 817-832.

Kobayashi, Y., Tomida, Y., Kamei, T. & Eguchi, T. 2006. Anatomy of a Japanese tomistomine crocodylian, *Toyotamaphimeia machikanensis* (Kamei et Matsumoto, 1965),

from the Middle Pleistocene of Osaka Prefecture: the reassessment of its phylogenetic status within Crocodylia. National Science Museum Monographs 35, 1-121.

Li, C., Wu, X.-C., Cheng, Y.-N., Sato, T. & Wang, L. 2006. An unusual new archosaurian from the marine Triassic of China.Naturwissenschaften 93, 200-206.

Lu, J.-C. & Ji, Q. 2006. Preliminary results of a phylogenetic analysis of the pterosaurs from western Liaoning and surrounding areas. Journal of the Paleontological Society of Korea 22, 239-261.

Martill, D. M. & Naish, D. 2006. Cranial crest development in the azhdarcoid pterosaur *Tupuxuara*, with a review of the genus and tapejarid monophyly. Palaeontology 49, 925-941.

McAliley, L. R., Willis, R. E., Ray, D. A., White, P. S., Brochu, C. A. & Densmore, L. D. 2006. Are crocodiles really monophyletic? - evidence for subdivisions from sequence and morphological data. Molecular Phylogenetics and Evolution 39, 16-32.

Parker, W. G. & Irmis, R. B. 2006. A new species of the Late Triassic phytosaur *Pseudopalatus* (Archosauria: Pseudoscuhia) from Petrified Forest National Park, Arizona. Museum of Northern Arizona Bulletin 62, 126-144.

Piras, P. and Buscalioni, A. D. 2006. *Diplocynodon muelleri comb. nov.*, an Oligocene diplocynodontine alligatoroid from Catalonia (Ebro Basin, Lleida Province, Spain). Journal of Vertebrate Paleontology 26, 608-620.

Renesto, S. & Binelli, G. 2006. *Vallesaurus cenensis* Wild, 1991, a drepanosaurid (Reptilia, Diapsida) fromthe Late Triassic of northern Italy. Rivista Italiana di Paleontologia e Stratigrafia 112, 77-94.

Salisbury, S. W., Molnar, R. E., Frey, E. & Willis, P. M. A. 2006. The origin of modern crocodyliforms: new evidence from the Cretaceous of Australia. Proceedings of the Royal Society of London B 273, 2439-2448.

Turner, A. H. 2006. Osteology and phylogeny of a new species of *Araripesuchus* (Crocodyliformes: Mesoeucrocodylia) from the Late Cretaceous of Madagascar. Historical Biology 18, 255-369.

Zaher, H., Pol, D., Carvalho, A. B., Riccomini, C., Campos, D. & Nava, W. 2006. Redescription of the cranial morphology of *Mariliasuchus amarali,* and its phylogenetic affinities (Crocodyliformes, Notosuchia). American Museum Novitates 3512, 1-40.

**2005**

Company, J., Pereda Suberbiola, X., Ruiz-Omenaca, J. I. and Buscalioni, A. D. 2005. A new species of *Doratodon* (Crocodyliformes: Ziphosuchia) from the Late Cretaceous of Spain. Journal of Vertebrate Paleontology 25, 343-353.

Delfino, M., Piras, P. & Smith, T. 2005. Anatomy and phylogeny of the gavialoid crocodylian *Eosuchus lerichei* from the Paleocene of Europe. Acta Palaeontologica Polonica 50, 565-580.

Evans, S. E. & Klembara, J. 2005. A choristoderan reptile (Reptilia: Diapsida) from the Lower Miocene of Northwest Bohemia (Czech Republic). Journal of Vertebrate Paleontology 25, 171-184.

Gao, K.-Q. & Fox, R. C. 2005. A new choristodere (Reptilia: Diapsida) from the Lower Cretaceous of western Liaoning Province, China, and phylogenetic relationships of Monjurosuchidae. Zoological Journal of the Linnean Society 145, 427-444.

Jouve, S. 2005. A new description of the skull of Dyrosaurus phosphaticus (Thomas, 1893) (Mesoeucrocodylia: Dyrosauridae) from the Lower Eocene of North Africa. Canadian Journal of Earth Sciences 42, 323-337.

Jouve, S., Iarochene, M., Bouya, B. & Amaghzaz, M. 2005. A new dyrosaurid crocodyliform from the Palaeocene of Morocco and a phylogenetic analysis of Dyrosauridae. Acta Palaeontologica Polonica 50, 581-594.

Ksepka, D. T., Gao, K.-Q. & Norell, M. A. 2005. A new choristodere from the Cretaceous of Mongolia. American Museum Novitates 3468, 1-22.

Mueller-Towe, I. J. 2005. Phylogenetic relationships of the Thalattosuchia. Zitteliana A45, 211-213.

Pol, D. & Apesteguia, S. 2005. New *Araripesuchus* remains from the early Late Cretaceous (Cenomanian-Turonian) of Patagonia. American Museum Novitates 3490, 1-38.

Turner, A. H. and Calvo, J. O. 2005. A new sebecosuchian crocodyliform from the Late Cretaceous of Patagonia. Journal of Vertebrate Paleontology 25, 87-98.

Wang, X.-L., Kellner, A. W. A., Zhou, Z.-H. and Campos, D. A. 2005. Pterosaur diversity and faunal turnover in Cretaceous terrestrial ecosystems in China. Nature 437, 875-879.

**2004**

Brochu, C. A. 2004. A new Late Cretaceous gavialoid crocodylian from eastern North America and the phylogenetic relationships of thoracosaurs. Journal of Vertebrate Paleontology 24, 610-633.

Brochu, C. A. 2004. Alligatorine phylogeny and the status of *Allognathosuchus* Mook, 1921. Journal of Vertebrate Paleontology 24, 857-873.

Brochu, C. A. & Rincon, A. D. 2004. A gavialoid crocodylian from the Lower Miocene of Venezuela. Special Papers in Palaeontology 71, 61-79.

Carvalho, I. S., Ribeiro, L. C. B. & Avilla, L. S. 2004. *Uberabasuchus terrificus sp. nov.,* a new crocodylomorpha from the Bauru Basin (Upper Cretaceous), Brazil. Gondwana Research 7, 975-1002.

Clark, J. M., Xu, X., Forster, C. A. & Wang, Y. 2004. A Middle Jurassic 'sphenosuchian' from China and the origin of the crocodylian skull. Nature 430, 1021-1024.

Gatesy, J., Baker, R. H. & Hayashi, C. 2004. Inconsistencies in arguments for the supertree approach: supermatrices versus supertrees of Crocodylia. Systematic Biology 53, 342-355.

Hua, S. & Jouve, S. 2004. A primitive marine gavialoid from the Paleocene of Morocco. Journal of Vertebrate Paleontology 24, 341-350.

Kellner, A. W. A. 2004. New information on the Tapejaridae (Pterosauria, Pterodactyloidea) and discussion of the relationships of this clade. Ameghiniana 41, 521-534.

Li, C., Rieppel, O. & LaBarbera, M. C. 2004. A Triassic aquatic protorosaur with an extremely long neck. Science 305, 1931.

Maisch, M. W., Matzke, A. T. & Sun, G. 2004. A new dsungaripteroid pterosaur from the Lower Cretaceous of the southern Junggar Basin, north-west China. Cretaceous Research 25, 625-634.

Modesto, S. P. & Sues, H.-D. 2004. The skull of the Early Triassic archosauromorph reptile *Prolacerta broomi* and its phylogenetic significance. Zoological Journal of the Linnean Society 140, 335-351.

Muller, J., 2004. The relationships among diapsid reptiles and the influence of taxon selection. In Arratia, G., Wilson, M. V. H. and Cloutier, R. (eds.) Recent Advances in the Origin and Early Radiation of Vertebrates. Veerlag Dr Friedrich Pfeil, Munchen, p379-408.

Pol, D. & Norell, M. A. 2004. A new gobiosuchid crocodyliform taxon from the Cretaceous of Mongolia. American Museum Novitates 3458, 1-31.

Pol, D. & Norell, M. A. 2004. A new crocodyliform from Zos Canyon, Mongolia. American Museum Novitates 3445, 1-36.

Pol, D., Ji, S.-A., Clark, J. M. & Chiappe, L. M. 2004. Basal crocodyliforms from the Lower Cretaceous Tugulu Group (Xinjiang, China), and the phylogenetic position of Edentosuchus. Cretaceous Research 25, 603-622.

Senter, P. 2004. Phylogeny of Drepanosauridae (Reptilia: Diapsida). Journal of Systematic Palaeontology 2, 257-268.

Turner, A. H. 2004. Crocodyliform biogeography during the Cretaceous: evidence of Gondwanan vicariance from biogeographical analysis. Proceedings of the Royal Society of London B 271, 2003-2009.

**2003**

Brochu, C. A. 2003. Phylogenetic approaches toward crocodylian history. Annual Review of Earth and Planetary Sciences 31, 357-397.

Gatesy, J., Amato, G., Norell, M. A., DeSalle, R. & Hayashi, C. 2003. Combined support for wholesale taxic atavism in gavialine crocodylians. Systematic Biology 52, 403-422.

Harris, S. R., Gower, D. J. & Wilkinson, M. 2003. Intraorganismal homology, character construction, and the phylogeny of aetosaurian archosaurs (Reptilia, Diapsida). Systematic Biology 52, 239-252.

Harshman, J., Huddleston, C. J., Bollback, J. P., Parsons, T. J. & Braun, M. J. 2003. True and false gharials: a nuclear gene phylogeny of Crocodylia. Systematic Biology 52, 386-402.

Kellner, A. W. A. 2003. Pterosaur phylogeny and comments on the evolutionary history of the group. Geological Society of London Special Publications 217, 105-137.

Martinelli, A. G. 2003. New cranial remains of the bizarre notosuchid *Comahuesuchus brachybuccalis* (Archosauria, Crocodyliformes) from the Late Cretaceous of Rio Negro Province (Argentina). Ameghiniana 40, 559-572.

Nesbitt, S. J. 2003. *Arizonasaurus* and its implications for archosaur divergence. Proceedings of the Royal Society of London B 270, S234-S237.

Pol, D. 2003. New remains of *Sphagesaurus huenei* (Crocodylomorpha: Mesoeucrocodylia) from the Late Cretaceous of Brazil.Journal of Vertebrate Paleontology 23, 817-831.

Rieppel, O., Fraser, N. C. & Nosotti, S. 2003. The monophyly of Protorosauria (Reptilia, Archosauromorpha): a preliminary analysis. Atti della Societa Italiana di Scienze Naturali e del Museo Civico di Storia Naturale di Milano 144, 359-382.

Rogers, J. V. 2003. *Pachycheilosuchus trinquei*, a new procoelous crocodyliform from the Lower Cretaceous (Albian) Glen Rose Formation of Texas. Journal of Vertebrate Paleontology 23, 128-145.

Sereno, P. C., Sidor, C. A., Larsson, H. C. E. and Gado, B. 2003. A new notosuchian from the Early Cretaceous of Niger. Journal of Vertebrate Paleontology 23, 477-482.

Sues, H.-D. 2003. An unusual new archosauromorph reptile from the Upper Triassic Wolfville Formation of Nova Scotia. Canadian Journal of Earth Sciences 40, 635-649.

Sues, H.-D., Olsen, P. E., Carter, J. G. & Scott, D. M. 2003. A new crocodylomorph archosaur from the Upper Triassic of North Carolina. Journal of Vertebrate Paleontology 23, 329-343.

Unwin, D. M. 2003. On the phylogeny and evolutionary history of pterosaurs. Geological Society of London Special Publications 217, 139-190.

Unwin, D. M. 2003. Eudimorphodon and the early history of pterosaurs. Rivista Museo civico Scienze Naturali "E. Caffi" Bergamo 22, 39-46.

Wu, X.-B., Wang, Y.-Q., Zhou, K.-Y., Zhu, W.-Q., Nie, J. & Wang, C.-L. 2003. Complete mitochondrial DNA sequence of Chinese alligator, *Alligator sinensis*, and phylogeny of crocodiles. Chinese Science Bulletin 48, 2050-2054.

**2002**

Benton, M. J. & Walker, A. D. 2002. *Erpetosuchus*, a crocodile-like basal archosaur from the Late Triassic of Elgin, Scotland.Zoological Journal of the Linnean Society 136, 25-47.

Brochu, C. A., Bouare, M. L., Sissoko, F., Roberts, E. M. and O'Leary, M. A. 2002. A dyrosaurid crocodyliform braincase from Mali. Journal of Paleontology 76, 1060-1071.

Clark, J. M. & Sues, H.-D. 2002. Two new basal crocodylomorph archosaurs from the Lower Jurassic and the monophyly of the Sphenosuchia. Zoological Journal of the Linnean Society 136, 77-95.

Gower, D. J. 2002. Braincase evolution in suchian archosaurs (Reptilia: Diapsida): evidence fom the rauisuchian *Batrachotomus kupferzellensis*. Zoological Journal of the Linnean Society 136, 49-76.

Hungerbuhler, A. 2002. The Late Triassic phytosaur *Mystriosuchus westphali*, with a revision of the genus. Palaeontology 45, 377-418.

Molnar, R. E., Worthy, T. & Willis, P. M. A., 2002. An extinct Pleistocene endemic mekosuchine crocodylian from Fiji. Journal of Vertebrate Paleontology, 22, 612-628.

Tykoski, R. S., Rowe, T. B., Ketcham, R. A. & Colbert, M. W. 2002. *Calsoyasuchus valliceps,* a new crocodyliform from the Early Jurassic Kayenta Formation of Arizona. Journal of Vertebrate Paleontology 22, 593-611.

**2001**

Brochu, C. A. 2001. Congruence between physiology, phylogenetics, and the fossil record on crocodylian historical biogeography. InG. Grigg, F. Seebacher and C. E. Franklin (eds.) Crocodilian Biology and Evolution. Surrey Beatty and Sons, Sydney, pp. 9-28.

Brochu, C. A. & Densmore, L. D., 2001. Crocodile phylogenetics: A review of current progress. In G. Grigg, F. Seebacher and C. E. Franklin (eds.) Crocodilian Biology and Evolution. Surrey Beatty and Sons, Sydney, p3-8.

Buscalioni, A. D., Ortega, E., Weishampel, D. B. & Jianu, C. M., 2001. A revision of the crocodyliform Allodaposuchus precedensfrom the Upper Cretaceous of the Hateg Basin, Romania. Its relevance in the phylogeny of Eusuchia. Journal of Vertebrate Paleontology, 21, 74-86.

Hungerbuhler, A. 2001. The status and phylogenetic relationships of *"Zanclodon" arenaceus*: the earliest known phytosaur?Palaeontologische Zeitschrift 75, 97-112.

Ray, D. A., White, P. S., Duong, H. V., Cullen, T. & Densmore, L. D. 2001. High levels of genetic variability in West African Dwarf Crocodiles *Osteolaemus tetraspis tetraspis*. In G. Grigg, F. Seebacher and C. E. Franklin (eds.) Crocodilian Biology and Evolution. Surrey Beatty and Sons, Sydney, p58-63.

Sereno, P. C., Larsson, H. C. E., Sidor, C. A. & Gado, B. 2001. The giant crocodyliform *Sarcosuchus* from the Cretaceous of Africa. Science 294, 1516-1519.

Wu, X.-C., Russell, A. P. & Cumbaa, S. L. 2001. *Terminonaris* (Archosauria: Crocodyliformes): new material from Saskatchewan, Canada, and comments on its phylogenetic relationships. Journal of Vertebrate Paleontology 21, 492-514.

**2000**

Brochu, C. A. 2000. Phylogenetic relationships and divergence timing of *Crocodylus* based on morphology and the fossil record. Copeia 3, 657-673.

Brochu, C. A. & Gingerich P. D., 2000. New tomistomine crocodylian from the Middle Eocene (Bartonian) of Wadi Hitan, Fayum Province, Egypt. Contributions from the Museum of Paleontology, The University of Michigan 30, 251-268.

Buckley, G. A., Brochu, C. A., Krause, D. W. & Pol, D. 2000. A pug-nosed crocodyliform from the Late Cretaceous of Madagascar. Nature 405, 941-944.

Clark, J. M., Sues, H.-D. & Berman, D. S. 2000. A new specimen of *Hesperosuchus agilis* from the Upper Triassic of New Mexico and the interrelationships of basal crocodylomorph archosaurs. Journal of Vertebrate Paleontology 20, 683-704.

Langer, M. C. & Schultz, C. L. 2000. A new species of the Late Triassic rhynchosaur *Hyperodapedon* from the Santa Maria Formation of South Brazil. Palaeontology 43, 633-652.

Larsson, H. C. E. & Gado, B. 2000. A new Early Cretaceous crocodyliform from Niger. Neues Jahrbuch fur Geologie und Palaontologie Abhandlungen 217, 131-141.

Olsen, P. E., Sues, H.-D. & Norell, M. A. 2000. First record of *Erpetosuchus* (Reptilia: Archosauria) from the Late Triassic of North America. Journal of Vertebrate Paleontology 20, 633-636.

Ortega, F., Gasparini, Z., Buscalioni, A. D. & Calvo, J. O. 2000. A new species of *Araripesuchus* (Crocodylomorpha, Mesoeucrocodylia) from the Lower Cretaceous of Patagonia (Argentina). Journal of Vertebrate Paleontology 20, 57-76.

Peters, D. 2000. A reexamination of four prolacertiforms with implications for pterosaur phylogenesis. Rivista Italiana di Paleontologia e Stratigrafia 106, 293-336.

**1999**

Brochu, C. A. 1999. Phylogenetics, taxonomy, and historical biogeography of Alligatoroidea. Journal of Vertebrate Paleontology 19, S9-S100.

Buckley, G. A. & Brochu, C. A. 1999. An enigmatic new crocodile from the Upper Cretaceous of Madagascar. Special Papers in Palaeontology 60, 149-175.

Evans, S. E. & Manabe, M. 1999. A choristodere reptile from the Lower Cretaceous of Japan. Special Papers in Palaeontology 60, 101-119.

Heckert, A. B. & Lucas, S. G. 1999. A new aetosaur (Reptilia: Archosauria) from the Upper Triassic of Texas and the phylogeny of aetosaurs. Journal of Vertebrate Paleontology 19, 50-68.

**1998**

Dilkes, D. W., 1998. The Early Triassic rhynchosaur *Mesosuchus browni* and the interrelationships of basal archosauromorph reptiles. Philosophical Transactions of the Royal Society of London B 353, 501-541.

Gao, K.-Q. & Fox, R. C. 1998. New choristoderes (Reptilia: Diapsida) from the Upper Cretaceous and Palaeocene, Alberta and Saskatchewan, Canada, and phylogenetic relationships of Choristodera. Zoological Journal of the Linnean Society 124, 303-353.

Trueman, J. W. H. 1998. Reverse successive weighting. Systematic Biology 47, 733-737.

**1997**

Benton, M. J. & Allen, J. L. 1997. *Boreopricea* from the Lower Triassic of Russia, and the relationships of the prolacertiform reptiles. Palaeontology 40, 931-953.

Brochu, C. A. 1997. Morphology, fossils, divergence timing, and the phylogenetic relationships of *Gavialis*. Systematic Biology 46, 479-522.

Brochu, C. A. 1997. A review of "*Leidyosuchus*" (Crocodyliformes, Eusuchia) from the Cretaceous through Eocene of North America. Journal of Vertebrate Paleontology 17, 679-697.

Gomani, E. M. 1997. A crocodyliform from the Early Cretaceous dinosaur beds, northern Malawi. Journal of Vertebrate Paleontology 17, 280-294.

Gower, D. J. & Sennikov, A. G. 1997. *Sarmatosuchus* and the early history of the Archosauria. Journal of Vertebrate Paleontology 17, 60-73.

Jalil, N.-E. 1997. A new prolacertiform diapsid from the Triassic of North Africa and the interrelationships of the Prolacertiformes. Journal of Vertebrate Paleontology 17, 506-525.

Willis, P. M. A. 1997. Review of fossil crocodilians from Australasia. Australian Zoologist 30, 287-298.

Wu, X.-C., Sues, H.-D. & Dong, Z.-M. 1997. *Sichuanosuchus shuhanensis*, a new ?Early Cretaceous protosuchian (Archosauria: Crocodyliformes) from Sichuan (China), and the monophyly of Protosuchia. Journal of Vertebrate Paleontology 17, 89-103.

**1996**

Bennett, S. C. 1996. The phylogenetic position of the Pterosauria within the Archosauromorpha. Zoological Journal of the Linnean Society 118, 261-308.

Gower, D. J. & Sennikov, A. G. 1996. Morphology and phylogenetic informativeness of early archosaur braincases. Palaeontology 39, 883-906.

Heckert, A. B., Hunt, A. P. & Lucas, S. G. 1996. Redescription of *Redondasuchus reseri*, a Late Triassicaetosaur (Reptilia: Archosauria) from New Mexico (U.S.A.), and the biochronology and phylogeny of aetosaurs. Geobios 29, 619-632.

Novas, F. E. 1996. Dinosaur monophyly. Journal of Vertebrate Paleontology 16, 723-741.

Ortega, F., Buscalioni, A. D. & Gasparini, Z. 1996. Reinterpretation and new denomination of *Atacisaurus crassiproratus* (Middle Eocene; Issel, France) as *cf. Iberosuchus* (Crocodylomorpha, Metasuchia). Geobios 29, 353-364.

Poe, S. 1996. Data set incongruence and the phylogeny of crocodilians. Systematic Biology 45, 393-414.

Salisbury, S. W. & Willis, P. M. A. 1996. A new crocodylian from the Early Eocene of south-eastern Queensland and a preliminary investigation of the phylogenetic relationships of crocodyloids. Alcheringa 20, 179-226.

Williamson, T. E. 1996. *?Brachychampsa sealeyi, sp nov.,* (Crocodylia, Alligatoroidea) from the Upper Cretaceous (lower Campanian) Menefee Formation, northwestern New Mexico. Journal of Vertebrate Paleontology 16, 421-431.

Wu, X.-C. & Sues, H.-D. 1996. Anatomy and phylogenetic relationships of *Chimaerasuchus paradoxus*, an unusual crocodyliform reptile from the Lower Cretaceous of Hubei, China. Journal of Vertebrate Paleontology 16, 688-702.

Wu, X.-C., Brinkman, D. B. & Russell, A. P. 1996. A new alligator from the Upper Cretaceous of Canada and the relationships of early eusuchians. Palaeontology 39, 351-375.

**1995**

Dilkes, D. W. 1995. The rhynchosaur *Howesia browni* from the Lower Triassic of South Africa. Palaeontology 38, 665-685.

Wu, X.-C., Sues, H.-D. & Sun, A. 1995. A plant-eating crocodyliform reptile from the Cretaceous of China. Nature 376, 678-680.

**1994**

Bennett, S. C. 1994. Taxonomy and systematics of the Late Cretaceous pterosaur *Pteranodon* (Pterosauria, Pterodactyloidea).Occasional Papers of the Museum of Natural History University of Kansas 169, 1-70.

Clark, J. M. 1994. Patterns of evolution in Mesozoic Crocodyliformes. In, Fraser, N. C. and Sues, H.-D. (eds.), In the Shadow of the Dinosaurs. Cambridge University Press, Cambridge p84-97.

Parrish, J. M. 1994. Cranial osteology of *Longosuchus meadei* and the phylogeny and distribution of the Aetosauria. Journal of Vertebrate Paleontology 14, 196-209.

Sereno, P. C. & Arcucci, A. B. 1994. Dinosaurian precursors from the Middle Triassic of Argentina: *Marasuchus lilloensis, gen. nov.* Journal of Vertebrate Paleontology 14, 53-73.

Sereno, P. C. & Arcucci, A. B. 1994. Dinosaurian precursors from the Middle Triassic of Argentina: *Lagerpeton chanarensis*. Journal of Vertebrate Paleontology 14, 385-399.

Wu, X.-C., Li, J.-L. & Li, X.-M. 1994. Phylogenetic relationship of *Hsisosuchus*. Vertebrata PalAsiatica 32, 166-180.

Wu, X.-C., Brinkman, D. B. & Lu, J.-C. 1994. A new species of *Shantungosuchus* from the Lower Cretaceous of Inner Mongolia (China), with comments on *S. chuhsienensis* Young, 1961 and the phylogenetic position of the genus. Journal of Vertebrate Paleontology 14, 210-229.

**1993**

Gasparini, Z., Fernandez, M. & Powell, J. 1993. New Tertiary sebecosuchians (Crocodylomorpha) from South America: phylogenetic implications. Historical Biology 7, 1-19.

Gatesy, J., DeSalle, R. & Wheeler, W. 1993. Alignment-ambiguous nucleotide sites and the exclusion of systematic data. Molecular Phylogenetics and Evolution 2, 152-157.

Parrish, J. M. 1993. Phylogeny of the Crocodylotarsi, with reference to archosaurian and crurotarsan monophyly. Journal of Vertebrate Paleontology 13, 287-308.

Wu, X-.C. & Brinkman, D. B. 1993. A new crocodylomorph of "mesosuchian" grade from the Upper Cretaceous Upper Milk River Formation, southern Alberta. Journal of Vertebrate Paleontology 13, 153-160.

Wu, X.-C. & Chatterjee, S. 1993. Dibothrosuchus elaphros, a crocodylomorph from the Lower Jurassic of China and the phylogeny of the Sphenosuchina. Journal of Vertebrate Paleontology 13, 58-89.

**1992**

Gatesy, J. & Amato, G. D. 1992. Sequence similarity of 12S ribosomal segment of mitochondrial DNAs of gharial and false gharial. Copeia 1992, 241-243.

Hecht, M. K. 1992. A new choristodere (Reptilia, Diapsida) from the Oligocene of France: an example of the Lazarus Effect. Geobios 25, 115-131.

Parrish, J. M. 1992. Phylogeny of the Erythrosuchidae (Reptilia: Archosauriformes). Journal of Vertebrate Paleontology 12, 93-102.

Sereno, P. C. & Wild, R. 1992. *Procompsognathus*: theropod, "thecodont" or both? Journal of Vertebrate Paleontology 12, 435-458.

**1991**

Densmore, L. D. & White, P. S. 1991. The systematics and evolution of the crocodile as suggested by restriction endonuclease analysis of mitochondrial and nuclear ribosomal DNA. Copeia 1991, 602-615.

Gasparini, Z., Chiappe, L. M. & Marta Fernandez, M. 1991. A new Senonian peirosaurid (Crocodylomorpha) from Argentina and a synopsis of the South American Cretaceous crocodilians. Journal of Vertebrate Paleontology 11, 316-333.

Parrish, J. M. 1991. A new specimen of an early crocodylomorph (*cf. Sphenosuchus sp.*) from the Upper Triassic Chinle Formation of Petrified Forest National Park, Arizona. Journal of Vertebrate Paleontology 11, 198-212.

Sereno, P. C. 1991. Basal archosaurs: phylogenetic relationships and functional implications. SVP Memoir 2, 1-53.

**1990**

Benton, M. J. 1990. The species of *Rhynchosaurus*, a rhynchosaur (Reptilia, Diapsida) from the Middle Triassic of England. Philosophical Transactions of the Royal Society of London B 328, 213-306.

Evans, S. E. 1990. The skull of *Cteniogenys*, a choristodere (Reptilia: Archosauromorpha) from the Middle Jurassic of Oxfordshire. Zoological Journal of the Linnean Society 99, 205-237.

Norell, M. A. & Clark, J. M. 1990. A reanalysis of *Bernissartia jagesii,* with comments on its phylogenetic position and its bearing on the origin and diagnosis of the Eusuchia. Bulletin de l'Institut Royal des Sciences Naturelles de Belqique, Sciences de la Terre 60, 115-128.

Sereno, P. C. & Arcucci, A. B. 1990. The monophyly of crurotarsal archosaurs and the origin of bird and crocodile ankle joints. Neues Jahrbuch fur Geologie und Palaontologie Abhandlungen 180, 21-52.

**1989**

Bennett, S. C. 1989. A pteranodontid pterosaur from the Early Cretaceous of Peru, with comments on the relationships of Cretaceous pterosaurs. Journal of Paleontology 63, 669-677.

Norell, M. A. 1989. The higher level relationships of the extant Crocodylia. Journal of Herpetology 23, 325-335.

**1988**

Benton, M. J. & Clark, J. M. 1988. Archosaur phylogeny and the relationships of the Crocodylia. In Benton, M. J. (ed.) The Phylogeny and Classification of the Tetrapods, Volume 1: Amphibians, Reptiles, Birds. Clarendon Press, Oxford, p295-338.

Buscalioni, A. D. & Sanz, J. L. 1988. Phylogenetic relationships of the Atoposauridae (Archosauria, Crocodylomorpha). Historical Biology 1, 233-250.

Gauthier, J., Kluge, A. G. & Rowe, T. 1988. Amniote phylogeny and the importance of fossils. Cladistics 4, 105-209.

Gauthier, J. A., Kluge, A. G. & Rowe, T. 1988. The early evolution of the Amniota. In M. J. Benton (ed.) The Phylogeny and Classification of the Tetrapods, Volume 1: Amphibians, Reptiles, Birds. Clarendon Press, Oxford, p103-155.
