# Supplementary material for: Decoupling speciation and extinction reveals both abiotic and biotic drivers shaped 250 million years of diversity in crocodile-line archosaurs
Source: Nat Ecol Evol. 2023 Dec 4;8(1):121–32. doi: 10.1038/s41559-023-02244-0 (PMC10781641; doi:10.1038/s41559-023-02244-0)
Supplement: Supplementary file 2 — Reporting Summary [file 41559_2023_2244_MOESM2_ESM.pdf]

nature portfolio

Corresponding author(s): *Double-anonymous peer review submissions: write DAPR and your manuscript number here instead of author names.*

Last updated by author(s): *YYYY-MM-DD*

# Reporting Summary

Nature Portfolio wishes to improve the reproducibility of the work that we publish. This form provides structure for consistency and transparency in reporting. For further information on Nature Portfolio policies, see our Editorial Policies and the Editorial Policy Checklist.

## Statistics

For all statistical analyses, confirm that the following items are present in the figure legend, table legend, main text, or Methods section.

| n/a | Confirmed | |
|---|---|---|
| ☐ | ☒ | The exact sample size (*n*) for each experimental group/condition, given as a discrete number and unit of measurement |
| ☒ | ☐ | A statement on whether measurements were taken from distinct samples or whether the same sample was measured repeatedly |
| ☐ | ☒ | The statistical test(s) used AND whether they are one- or two-sided<br>*Only common tests should be described solely by name; describe more complex techniques in the Methods section.* |
| ☒ | ☐ | A description of all covariates tested |
| ☒ | ☐ | A description of any assumptions or corrections, such as tests of normality and adjustment for multiple comparisons |
| ☐ | ☒ | A full description of the statistical parameters including central tendency (e.g. means) or other basic estimates (e.g. regression coefficient) AND variation (e.g. standard deviation) or associated estimates of uncertainty (e.g. confidence intervals) |
| ☐ | ☒ | For null hypothesis testing, the test statistic (e.g. *F*, *t*, *r*) with confidence intervals, effect sizes, degrees of freedom and *P* value noted<br>*Give P values as exact values whenever suitable.* |
| ☐ | ☒ | For Bayesian analysis, information on the choice of priors and Markov chain Monte Carlo settings |
| ☒ | ☐ | For hierarchical and complex designs, identification of the appropriate level for tests and full reporting of outcomes |
| ☒ | ☐ | Estimates of effect sizes (e.g. Cohen's *d*, Pearson's *r*), indicating how they were calculated |

*Our web collection on statistics for biologists contains articles on many of the points above.*

## Software and code

Policy information about availability of computer code

| | |
|---|---|
| Data collection | Phlyogenetic and ecological data were obtained from the literature, data on fossil age ranges were obtained from the Paleobiology database (paleobiodb.org). |
| Data analysis | TNT 1.5 was used to analyse the phylogenetic data, FossilBAMM was used to obtain the diversification dynamics, BAMMtools 2.1.7 was used to analyse the FossilBAMM output. All analyses were carried out in R 4.1.2. |

For manuscripts utilizing custom algorithms or software that are central to the research but not yet described in published literature, software must be made available to editors and reviewers. We strongly encourage code deposition in a community repository (e.g. GitHub). See the Nature Portfolio guidelines for submitting code & software for further information.

## Data

Policy information about availability of data

All manuscripts must include a data availability statement. This statement should provide the following information, where applicable:

- Accession codes, unique identifiers, or web links for publicly available datasets
- A description of any restrictions on data availability
- For clinical datasets or third party data, please ensure that the statement adheres to our policy

All underlying data needed to replicate the results are available in the supplementary files. Data generated by the analyses are available from the corresponding author (KED) on reasonable request.

## Research involving human participants, their data, or biological material

Policy information about studies with human participants or human data. See also policy information about sex, gender (identity/presentation), and sexual orientation and race, ethnicity and racism.

| | |
|---|---|
| Reporting on sex and gender | NA |
| Reporting on race, ethnicity, or other socially relevant groupings | NA |
| Population characteristics | NA |
| Recruitment | NA |
| Ethics oversight | NA |

Note that full information on the approval of the study protocol must also be provided in the manuscript.

# Field-specific reporting

Please select the one below that is the best fit for your research. If you are not sure, read the appropriate sections before making your selection.

☐ Life sciences ☐ Behavioural & social sciences ☒ Ecological, evolutionary & environmental sciences

For a reference copy of the document with all sections, see nature.com/documents/nr-reporting-summary-flat.pdf

# Ecological, evolutionary & environmental sciences study design

All studies must disclose on these points even when the disclosure is negative.

| | |
|---|---|
| Study description | We build a new phylogenetic tree of crocodile-line archosaurs and use it to model diversification dynamics through their evolutionary history. We partition our data set by ecology to explore the effects on ecology on macroevolutionary responses to climate change and biotic interactions. We show that both abiotic (temperature and sea level) and biotic (niche filling/competiton) variables drove diversification in crocodile-line archosaurs over their 250 million year history but that the effects are ecology dependent. |
| Research sample | Our raw data consist of phylogenetic trees collected from the literature. The underlying data were reanalysed to build our phylogeny, which is fundamental to our analyses. |
| Sampling strategy | We collected all phylogenetic data containing crocodile-line archosaurs with no exclusions. |
| Data collection | Phylogenetic data were collected by GTL from the literature. Ecological and fossil age data were collected by ARDP and PDM from the literature and from the paleobiology database. |
| Timing and spatial scale | NA - All published data were collected. |
| Data exclusions | NA |
| Reproducibility | NA - All analyses are computational in nature and the results can be replicated. |
| Randomization | NA - randomisation is not relevant to the nature of the data or the type of study. |
| Blinding | NA - blinding is not relevant to the nature of the data or the type of study. |

Did the study involve field work? ☐ Yes ☒ No

# Reporting for specific materials, systems and methods

We require information from authors about some types of materials, experimental systems and methods used in many studies. Here, indicate whether each material, system or method listed is relevant to your study. If you are not sure if a list item applies to your research, read the appropriate section before selecting a response.

## Materials & experimental systems

| n/a | Involved in the study |
|---|---|
| ☒ | ☐ Antibodies |
| ☒ | ☐ Eukaryotic cell lines |
| ☒ | ☐ Palaeontology and archaeology |
| ☒ | ☐ Animals and other organisms |
| ☒ | ☐ Clinical data |
| ☒ | ☐ Dual use research of concern |
| ☒ | ☐ Plants |

## Methods

| n/a | Involved in the study |
|---|---|
| ☒ | ☐ ChIP-seq |
| ☒ | ☐ Flow cytometry |
| ☒ | ☐ MRI-based neuroimaging |
